# Infection with acanthocephalans increases tolerance of *Gammarus roeselii* (Crustacea: Amphipoda) to pyrethroid insecticide deltamethrin

**DOI:** 10.1007/s11356-023-26193-0

**Published:** 2023-03-10

**Authors:** Judith Kochmann, Melanie Laier, Sven Klimpel, Arne Wick, Uwe Kunkel, Jörg Oehlmann, Jonas Jourdan

**Affiliations:** 1 Department of Integrative Parasitology and Zoophysiology, Goethe University of Frankfurt, Max-von-Laue-Straße 13, D-60438 Frankfurt am Main, Germany; 2 Senckenberg Biodiversity and Climate Research Centre, Senckenberganlage 25, D-60325 Frankfurt am Main, Germany; 3 Johannes Gutenberg University Mainz, Hanns-Dieter-Hüsch Weg 15, 55128 Mainz, Germany; 4 Department Aquatic Ecotoxicology, Goethe University of Frankfurt, Max-von-Laue-Straße 13, D-60438 Frankfurt am Main, Germany; 5 Federal Institute of Hydrology, Am Mainzer Tor 1, D-56068 Koblenz, Germany; 6 Present Address: Bavarian Environment Agency, Specific Analysis for Environmental Monitoring, Bürgermeister-Ulrich-Str. 160, D-86179 Augsburg, Germany

**Keywords:** Chemical pollution, *Pomphorhynchus laevis*, *Polymorphus minutus*, Acute toxicity, Parasite prevalence, Pollution gradient, Pesticide, Wastewater treatment

## Abstract

Crustacean amphipods serve as intermediate hosts for parasites and are at the same time sensitive indicators of environmental pollution in aquatic ecosystems. The extent to which interaction with the parasite influences their persistence in polluted ecosystems is poorly understood. Here, we compared infections of *Gammarus roeselii* with two species of Acanthocephala, *Pomphorhynchus laevis*, and *Polymorphus minutus*, along a pollution gradient in the Rhine-Main metropolitan region of Frankfurt am Main, Germany. Prevalence of *P. laevi*s was very low at the unpolluted upstream reaches (*P* ≤ 3%), while higher prevalence (*P* ≤ 73%) and intensities of up to 9 individuals were found further downstream—close to an effluent of a large wastewater treatment plant (WWTP). Co-infections of *P. minutus* and *P. laevis* occurred in 11 individuals. Highest prevalence of *P. minutus* was *P* ≤ 9% and one parasite per amphipod host was the maximum intensity recorded. In order to assess whether the infection affects survival in the polluted habitats, we tested the sensitivity of infected and uninfected amphipods towards the pyrethroide insecticide deltamethrin. We found an infection-dependent difference in sensitivity within the first 72 h, with an effect concentration (24 h EC_50_) of 49.8 ng/l and 26.6 ng/l for infected and uninfected *G. roeselii*, respectively. Whereas final host abundance might partially explain the high prevalence of *P. laevis* in *G. roeselii*, the results of the acute toxicity test suggest a beneficial effect of acanthocephalan infection for *G. roeselii* at polluted sites. A strong accumulation of pollutants in the parasite could serve as a sink for pesticide exposure of the host. Due to the lack of a co-evolutionary history between parasite and host and a lack of behavioral manipulation (unlike in co-evolved gammarids), the predation risk by fish remains the same, explaining high local prevalence. Thus, our study exemplifies how organismic interaction can favor the persistence of a species under chemical pollution.

**Supplementary Information:**

The online version contains supplementary material available at 10.1007/s11356-023-26193-0.

## Introduction

The steadily increasing production of synthetic chemicals and their input into the environment poses a great burden on our ecosystems (Bernhardt et al. 2017). Very recently, Persson et al. (2022) defined the risk of chemical pollution and warned that the safe operating space of the planetary boundary is exceeded since annual production and releases are increasing at a pace that outstrips the global capacity for assessment and monitoring. Freshwater ecosystems are particularly affected by the accumulation of numerous chemical substances as they are the lowest points in the landscape. In addition to chemical loads, morphological degradation, as well as biotic stressors such as predation, invasive species or parasites, make streams a multi-stressor environment (Birk et al. 2020). The interaction of these different types of stressors ultimately determines the structure of species communities (Jackson et al. 2016; Nõges et al. 2016). In urbanized regions, we often observe a gradual increase in anthropogenic pressures from a river’s source to downstream regions, accompanied by a change in species composition. Such changes are often described for fishes (Matthews 1998; Jackson et al. 2011; Jourdan et al. 2016) and arthropods (Piscart et al. 2005; Weigand et al. 2020), but rarely for parasites and even less often considered collectively (Gilbert and Avenant-Oldewage 2017; Sures et al. 2017).

Parasites are essential components of all aquatic ecosystems where they affect the structure of biodiversity and the food web (Poulin 1999; Marcogliese 2004). Acanthocephala are especially abundant in aquatic habitats and their life cycle involves at least two different hosts. First intermediate hosts are often crustaceans, e.g., amphipods. These intermediate hosts are required for the development of larval stages. Larval development of Acanthocephala involves three different stages. The first larval stage (acanthor) develops and is ingested by the intermediate host where it reaches its second developmental stage (acanthella) and finally the third stage (cystacanth). The third stage is the infectious stage for the final host (Kennedy 2006; Lucius et al. 2018). A final host is a host organism in which a parasite becomes sexually mature and reproduces (Zander 1998; Mehlhorn and Piekarski 2002; Kennedy 2006). The most common aquatic Acanthocephala in Europe are *Pomphorhynchus laevis*, *Pomphorhynchus tereticollis*, and *Polymorphus minutus* (Schmidt-Rhaesa 2015). Final hosts in the life cycle of Acanthocephala are vertebrates like fish (e.g., for *P. laevis*) or birds (e.g., for *P. minutus*). Some species can have negative impacts on their intermediate host species, e.g., *P. laevis* cystacanths can reduce growth and oxygen uptake, as well as egg production in *Gammarus pulex* (Kennedy 2006). On the other hand, a recent study provides evidence why infection could also be beneficial for amphipods: Rothe et al. (2022) explored the role of three biomarkers (phenoloxidase activity, glycogen, and lipid concentrations) in *G. fossarum* infected with *P. minutus* and found a higher lipid and glycogen content in infected *G. fossarum*.

The interaction of species and their parasites may be an essential component that explains the persistence or disappearance of species under multiple stress. Acanthocephalan parasites are known to accumulate numerous pollutants, such as toxic metals (Sures 2004; Nachev and Sures 2016) and selected organic pollutants (Yen et al. 2014), affecting biological availability of these substances. For example, the acanthocephalan parasite *Pomphorhynchus laevis* can rapidly accumulate lead, reaching concentrations of this metal that are significantly greater than in its final host *Squalius cephalus* (Sures et al. 1994; Sures and Siddall 1999; Molbert et al. 2020). Due to the high accumulation of pollutants, parasites can affect the pollutant metabolism of their hosts and thus act as a pollutant sink (Sures et al. 2017). Acanthocephalans have a complex life cycle; while adult stages live in the intestine of the final hosts, their infective larval stages (cystacanths) develop in the hemocoel of arthropod intermediate hosts, such as amphipod crustaceans. Here, the effects on the intermediate host are less clear. Some studies suggest lower metal accumulation in parasites and higher mortalities of infected intermediate hosts (Grabner and Sures 2019 and references therein). For example, the amphipod *Gammarus pulex* infected with *P. laevis* and exposed to cadmium or aluminium suffered about two-fold higher mortality compared to uninfected individuals (McCahon et al. 1988; McCahon and Poulton 1991; Frank et al. 2013). However, there are other reports suggesting that there may be species- and contaminant-specific differences, for example, the mortality of *Gammarus roeselii* infected with *Polymorphus minutus* was reduced compared to uninfected *G. roeselii*, when exposed to palladium (Sures and Radszuweit 2007) and tests on cadmium revealed contrasting mortality patterns in infected males and females of *G. roeselii* (Gismondi et al. 2012).

Amphipods are commonly used indicator species and are considered to be key components of riverine food webs, as their shredding activity accelerates leaf fragmentation, produces faecal pellets, and transfers nutrients into secondary production, all of which are vital for maintaining diverse aquatic food webs (Petersen and Cummins 1974; Graça, 2001; Dangles and Malmqvist 2004). In the last century, a major restructuring of amphipod species communities has occurred in Central Europe, with non-native species displacing native species in many places, especially in the large river systems (Leuven et al. 2009). Because the middle and lower reaches of rivers are often more contaminated with chemical substances, depending on the surrounding land use with the input of pollutants from agriculture or from WWTPs (Munz et al. 2017; Beckers et al. 2018), a greater tolerance towards pollution can be assumed for invading species. Some empirical studies confirm this assumption (Bundschuh et al. 2013). Our study species, *G. roeselii* actually represents a species complex from which one genetic lineage has spread over Central Europe in the last centuries, probably facilitated by a combination of anthropogenic activities and natural range expansion (Jażdżewski 1980; Csapó et al. 2020). Due to a high adaptive capacity, they are now found in many waters of Central Europe, often also in highly polluted regions (Jourdan et al. 2019). Non-native species often have a lower parasite load (Torchin et al. 2003), which improves fitness (such as fecundity and/or survival), promoting population growth and further spread. However, if they are exposed to and affected by new parasites, the advantages of the so-called enemy release can diminish over time (Prenter et al. 2004; Kelly et al. 2009).

In our present study, we examined variations in acanthocephalan infections (prevalence, abundance, intensities) of the amphipod *G. roeselii* along a river gradient that is under strong anthropogenic pressure (Brettschneider et al. 2019). Along this river gradient, *G. roeselii* occurs in high abundance and frequent observations of acanthocephalan parasites (cystacanth stage easily observable by its intense orange coloration) motivated us to analyze their distribution in relation to the local pollution load. Therefore, in a first, more descriptive part, we show the infection patterns in relation to local environmental conditions, while, in a second part, we investigate whether the infection with acanthocephalans influences the sensitivity of amphipods to anthropogenic stressors such as the commonly used insecticide deltamethrin (Lu et al. 2019). Here, we expected that acanthocephalan infection would result in lower tolerance of *G. roeselii* to deltamethrin.

## Material and methods

### Sampling

**Fig. 1:**
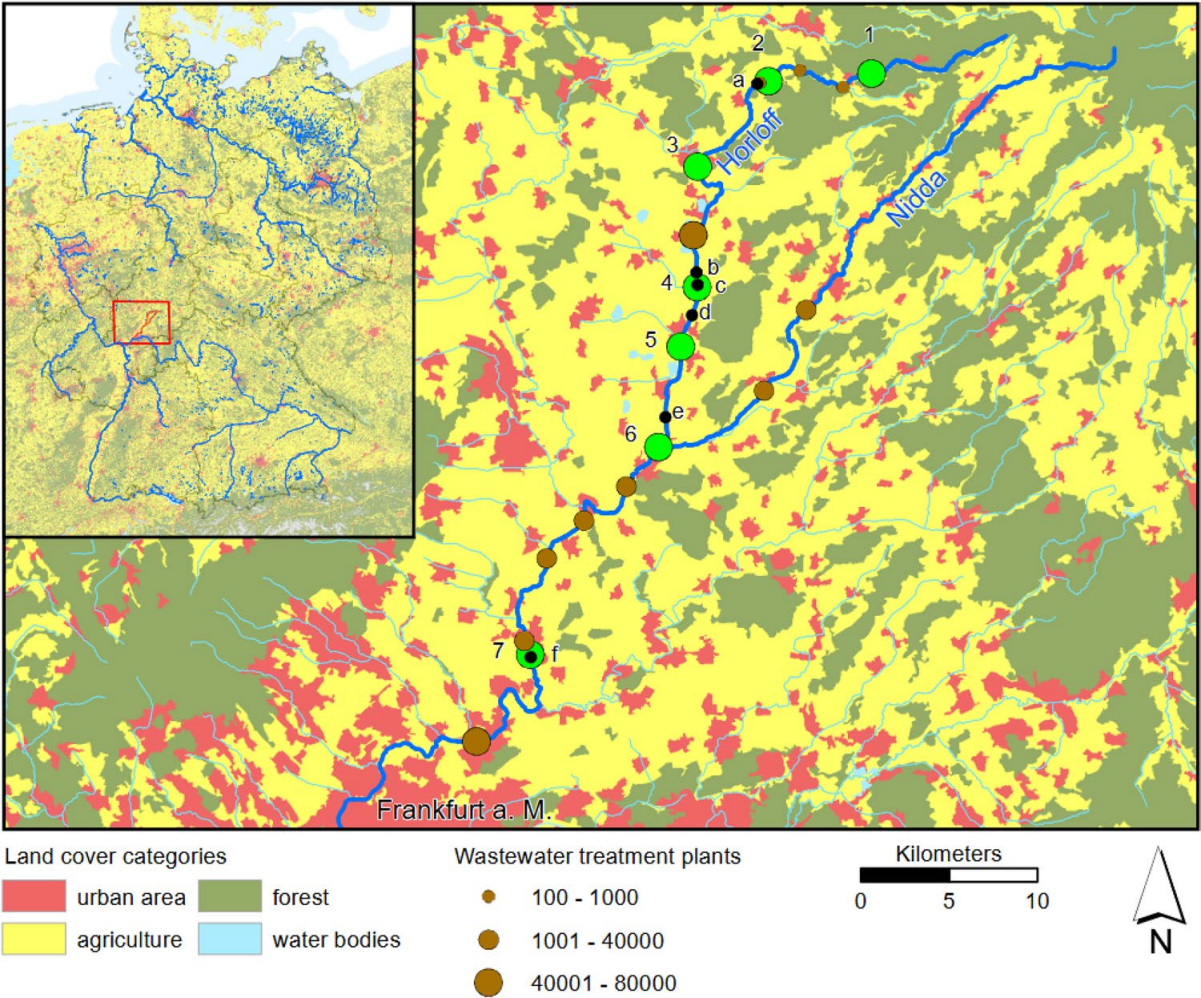
Map of the Nidda catchment in Hesse, Germany, with sampling sites 1–7 (green dots). Discharges from wastewater treatment plants are depicted by brown dots and are categorized according to the size of the plant (population equivalents). Information on fish fauna sampled at sites a-f (black dots) between 2007 and 2018 can be found in Appendix 1: Figure S1. Geographical coordinates can also be found in Appendix 1: Table S1. Information on land cover according to the EEA CLC2018 data, level 1 classification. Map generated in Esri ArcGIS version 10.8.2. Projection: ETRS 1989 UTM Zone 32N

We collected *Gammarus roeselii* from seven sites in the Nidda Basin (Hesse, Germany) ranging from the main river Nidda to a tributary, the Horloff river, which is less polluted in its upper course, but heavily anthropogenically polluted in its lower course (Hessian Agency for Nature Conservation, Environment and Geology; Brettschneider et al. 2019; Fig. 1). The Nidda basin is one of the most important surface waters in the Rhine-Main metropolitan region and covers an area of almost 2000 km^2^. We collected *G. roeselii* between 24th September 2020 and 5th October 2020. Amphipods were collected without any visual pre-inspection or pre-sorting by turning around stones and kick-sampling method covering all available habitats, using a hand net (0.5 mm mesh size). Samples were kept in aerated cooling boxes and were transported back to the laboratory where they were frozen at – 20 °C until further processing. A minimum number of 100 amphipods was sampled at each site (except for site 2) and the catch per unit effort (CPUE) was estimated as a proxy for relative amphipod abundance. CPUE was calculated for each sampling site and defined as individuals taken per person and unit time spent sampling.

### Parasite identification and quantification

**Fig. 2:**
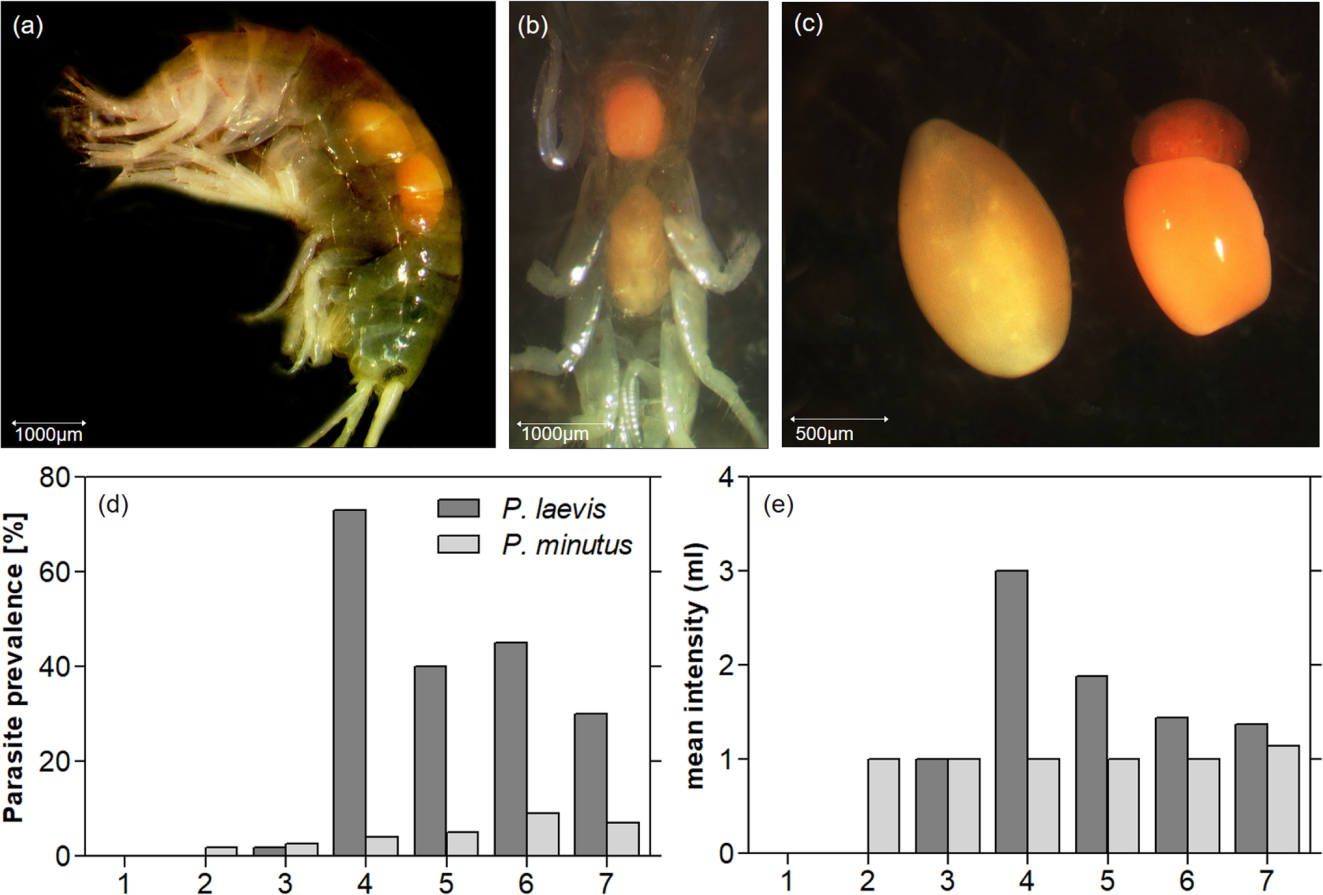
Co-infection of *Gammarus roeselii* with larval stages of *Pomphorhynchus laevis* and *P. minutus* (pictures) with **a** lateral view, **b** ventral view, and **c** isolated specimens of *P. laevis* (left) and *P. minutus* (right). The **d** prevalence and **e** mean intensity (mI) of *P. laevis* and *P. minutus* at the seven sampling sites (from most upstream site 1 to downstream site 7). Note that co-infections only occurred in 11 individuals

A total of n = 674 individuals of *G. roeselii* were examined morphologically under a stereomicroscope (OLYMPUS SZ61, Olympus Corporation, Tokyo, Japan), taking pictures of each individual with an integrated camera (JVC KY-F75U). Individuals were sexed and body size was measured from the anterior end of the rostrum to the end of the telson following the curved outline of the body (see Jourdan et al. 2019), using the software DISKUS (Version: 4.50.1458. Developer: Technisches Büro Hilgers, Königwinter, Germany). We then dissected the amphipods to carefully remove acanthocephalan parasites. Due to their conspicuous orange-red coloration, the parasites could often be spotted directly while observing the lateral or ventral side of the amphipod individual (Fig. 2). All parasites were photographed and frozen at – 20 °C.

We isolated and identified a total of *n* = 432 acanthocephalan parasites. For identification, we initially used information provided by Golvan (1969), Perrot-Minnot (2004) and Labaude (2016). Color and shape of the surface were the main morphological characteristics to differentiate the cystacanth stage of the two acanthocephalan species. While *P. minutus* appeared more round and intense in color with a rather smooth body surface, *P. laevis* appeared light orange, ribbed and slightly more elongated. We furthermore verified our—slightly objective—assessment of species identity, by using a molecular approach on a subset of individuals (*n* = 213). Therefore, we amplified a portion of the internal transcribed spacer (ITS) rDNA gene and identified individuals to species level. Based on the genetically identified individuals, we could confirm all morphologically identified individuals. For details on DNA extraction, amplification and sequencing see Appendix 2, Figure S3.

Parasite prevalence (P), mean abundance (mA), mean intensity (mI) and intensity (I) were calculated for each parasite species according to Bush et al. (1997). Prevalence (P) is the number of hosts infected with one or more individuals of a parasite species divided by the number of hosts examined. Minimum or maximum intensity (*I*_min_, *I*_max_) describes the number of individuals of a parasite species found in a single host specimen. Mean intensity (mI) is calculated as the total number of parasite individuals of a particular parasite species divided by the total number of hosts infected by at least one individual of this parasite species. Mean abundance (mA) describes the total number of individuals of a parasite species divided by the number of hosts examined.

### Environmental variables

We recorded various water parameters parallel to the biological sampling. At each site, we measured a range of basic water quality parameters (temperature, conductivity, pH, oxygen concentration) using a HACH® HQ40d multimeter. We further determined nitrite and nitrate content as well as carbonate and total hardness using MColortest kits (Merck, Germany). In our analyses, we also considered the location of WWTPs. Municipal WWTPs affect the water quality of the Nidda and its tributaries whereby the percentage of clearwater (i.e., treated wastewater) reaches up to 50% as mean value for the years 2011–2015, with even higher values reached in the summer months with little precipitation (Fuchs et al. 2018). Information on geographical coordinates of WWTPs can be found in Appendix 1, Table S1. Since many pressures on riverine ecosystems arise from agricultural land use and can cause land-use-related stress in aquatic invertebrates (Ohler et al. 2022), we have also considered land-use data. Therefore, we extracted the percentage (weighted average) of the agriculturally used area in the subcatchment at our seven sampling sites from the data set of Domisch et al. (2015). A major explanatory variable for the occurrence of acanthocephalans is the presence of final hosts. For this, we have obtained information on the fish fauna sampled between 2007 and 2018 from the Hessian Agency for Nature Conservation, Environment and Geology (Appendix 1, Figure S1).

### Chemical analysis and calculation of mixture effect (*E*_CA_)

We collected water samples for the chemical analysis as grab samples between July 2015 and July 2016 in eight sampling campaigns, covering all seasons. We analyzed water samples for 161 medium polar emerging pollutants, including transformation products: 54 pharmaceuticals and 53 transformation products (TPs), 18 biocides and pesticides with 9 TPs, 10 specific industrial compounds, 4 X-ray contrast media, 3 artificial sweeteners, 2 corrosion inhibitors and 2 TPs, 1 repellent with 3 TPs, 1 aversive agent, and 1 stimulant. These compounds were analyzed by liquid chromatography (Agilent 1260 infinity series) coupled to tandem mass spectrometry (Sciex Triple Quad 6500 +) (LC–MS/MS) according to a method published by Hermes et al. (2018). We selected the compounds based on their frequency of detection in the literature and their persistence in urban water cycles. They comprise already regulated priority pollutants (e.g., isoproturon, terbutryn, and diuron) and river basin specific pollutants (e.g., triclosan, mecoprop, terbuthylazine, metazachlor, metolachlor, imidacloprid, carbendazim, and propiconazole) as well as compounds of the 1st, 2nd, and 3rd Watch List under the Water Framework Directive (e.g., diclofenac, sulfamethoxazole, trimethoprim, venlafaxine with o-desmethylvenlafaxine, and fluconazole). Moreover, we selected compounds, such as the antiepileptic carbamazepine and the artificial sweetener sucralose as they are conservative tracers of municipal wastewater (Scheurer et al. 2011).

In order to determine the actual toxicity levels at our sample sites (except for site 7 due to the lack of analytical data), we first calculated toxic units (TUs, dimensionless, see formula 1) for 57 of the 161 analyzed compound with available toxicity data on the basis of the arithmetic mean value from all sampling campaigns (*C*_mean_, ng/l) and its 50% effect concentration for immobilization in acute tests with *Daphnia* spp. (EC_50_, mg/l). In July 2016, a high-resolution time series of concentration data from a 12-day sampling campaign with a sampling interval of 4 h was available at some sampling points. This data was used to calculate two 6 days mean values in order not to overweight the data from this special sampling campaign for the sampling site. In accordance to Appendix 9 of the German Surface Water Ordinance (2016), we replaced measured values below the limit of quantification by half the value of the limit of quantification. If the calculated annual mean concentration (*c*_mean_) was below the limit of quantification, this value is given in Appendix 1, Table S3 as “ < LOQ” (below the limit of quantification) and the respective TU value was set to zero in Table S4. Otherwise, we calculated the TU according to formula (1):

1 $$\text{TU } = \frac{ {\text{C}}_{\text{mean }}}{{\text{ EC}}_{50}}$$

The EC_50_ values were obtained from the US-EPA ECOTOX database (https://cfpub.epa.gov/ecotox/), registration dossiers of the European Chemicals Agency (https://echa.europa.eu/), the Pesticide Properties Database of the International Union of Pure and Applied Chemistry (http://sitem.herts.ac.uk/aeru/iupac/index.htm) or from the scientific peer-reviewed literature (see Appendix 1, Table S4 for details).

According to the concentration addition (CA) concept of Loewe and Muischnek (1926) the mixture effect (*E*_CA_) results from the sum of the effects of the individual substances *i* of a mixture of n substances, calculated as TUs (formula 2):

2 $${\text{E}}_{\text{CA}}\text{ } = \sum\nolimits_{\text{i =1}}^{\text{n}}{{\text{TU}}}_{\text{i}}$$

In the CA concept, the concentration of each individual substance in the mixture is scaled to its respective toxicity. Each component of the mixture thus contributes to the effect of the mixture depending on its concentration and effectiveness. The calculation of *E*_CA_ thus allows to assess the relative toxicity in a water sample in comparison to other samples on the basis of the measured concentrations of contaminants. There is a time difference of 4 to 5 years between chemical and biological sampling. However, neither the use of the surrounding land in the study area nor the technology used in the WWTPs changed during this period, so that it can be assumed that the contamination profile along the investigated river stretch remained the same.

### Correlation analysis

We conducted several Pearson correlations to test for linear relationships. First, we correlated the size of amphipods and the number of parasites, and second, we related the prevalence (P) of *P. laevis* and *P. minutus* to environmental variables. Correlation matrices were visualized as a correlation plot using the *corrplot* function ('corrplot' package; Wei and Simko 2021) in R (R Core Team 2022).

### Acute toxicity tests

To examine the vulnerability of infected and uninfected *G. roeselii* to an acute input of pesticides, we selected the insecticide deltamethrin as a representative of pyrethroid insecticides, a group of fast acting insecticides used in agriculture, forestry, healthcare, and veterinary medicine (Palmquist et al. 2012). Deltamethrin in particular is one of the most frequently used insecticides and acaricides worldwide (Lu et al. 2019). The primary target site for pyrethroids are the voltage-gated sodium channels of the nervous system. Pyrethroids impede the closing of the channels, thus altering nerve function to cause repetitive firing and exhaustion of the nerve cells. These effects manifest as incoordination, convulsions, and paralysis of the organism (Soderlund and Bloomquist 1989; Davies et al. 2007). Deltamethrin has been shown to be toxic to *Gammarus* even at very low concentrations (Adam et al. 2010; Grethlein et al. 2022) which motivated us to choose it as a model toxicant.

To evaluate whether infected *G. roeselii* have a higher tolerance to the insecticide deltamethrin, we collected adult *G. roeselii* in the middle reaches of the Horloff River (site 5) in November 2020. Individuals were again collected by kick-sampling and were transported to the institute at Goethe University Frankfurt in aerated cooling boxes. They were placed into an aquarium which was kept in a climatic chamber at 10 °C with a light/dark cycle of 16:8 h. Half the amount of water in the aquarium consisted of water from the sampling site and the other half of SAM-5S medium (Borgmann 1996). The water was gradually replaced over a few days by SAM-5S medium and several aquaria were used to separate amphipods with (‘infected’) and without (‘uninfected’) visible acanthocephalan parasite infection. The aquaria were kept well aerated and amphipods were fed every second day with alder leaves (*Alnus glutinosa*) and small amounts of Tetra Min food flakes over an acclimation period of 7 days.

To test the sensitivity of infected and uninfected *G. roeselii* towards the pyrethroid insecticide deltamethrin, a stock solution of 1 mg/l was set up. For this, 1 mg of deltamethrin was dissolved in 1 ml of dimethyl sulfoxide (DMSO) and diluted at 1:1000 with SAM-5S medium. Eight nominal test concentrations (12.5; 25; 37.5; 50; 75; 100; 150, and 200 ng/l) were prepared from the stock solution using SAM-5S medium. One hundred milliliters of beaker glasses were used as individual testing chambers and filled with 30 ml of each test concentration solution. One individual of either infected or uninfected *G. roeselii* was placed in one beaker. Ten replicates for infected and uninfected individuals were used for each test concentration and for the negative control (SAM-5S). For the solvent control (200 μl/l DMSO), only 5 replicates were used. Over the experimental period of 96 h, glass beakers were covered with a glass lid to prevent evaporation and amphipods were not fed.

The condition (‘mobile’, ‘immobile’, ‘dead’) of each amphipod was observed and recorded after 24 h, 48 h, 72 h, and 96 h by gently stirring each beaker for 30 s. Immobility was defined as lack of movement response when the test vessel was carefully swayed. In a few cases, *G. roeselii* individuals classified as immobile at a particular measurement time point were classified as mobile thereafter. In such cases, individual amphipods were consistently classified as mobile individuals, even if they appeared immobile before their apparent recovery. For the calculation of the EC_50_, the immobile and dead individuals were summed up.

The acute toxicity was expressed as median effective concentration (EC_50_), which was taken as the concentration that killed or immobilized 50% of the amphipods. We fitted dose–response models using non-linear parametric functions implemented in the ‘drc’ package in R (Ritz et al. 2016). Therefore, we used two parameter log–logistic models (LL.2) to analyze the acute toxicity test data for different exposure times and the two groups of amphipods (infected/uninfected). To statistically validate differences in the EC_50_ values of infected and uninfected amphipods, we used the *compParm* function on the fitted LL2 models.

## Results

### Acanthocephalan infection patterns

**Fig. 3:**
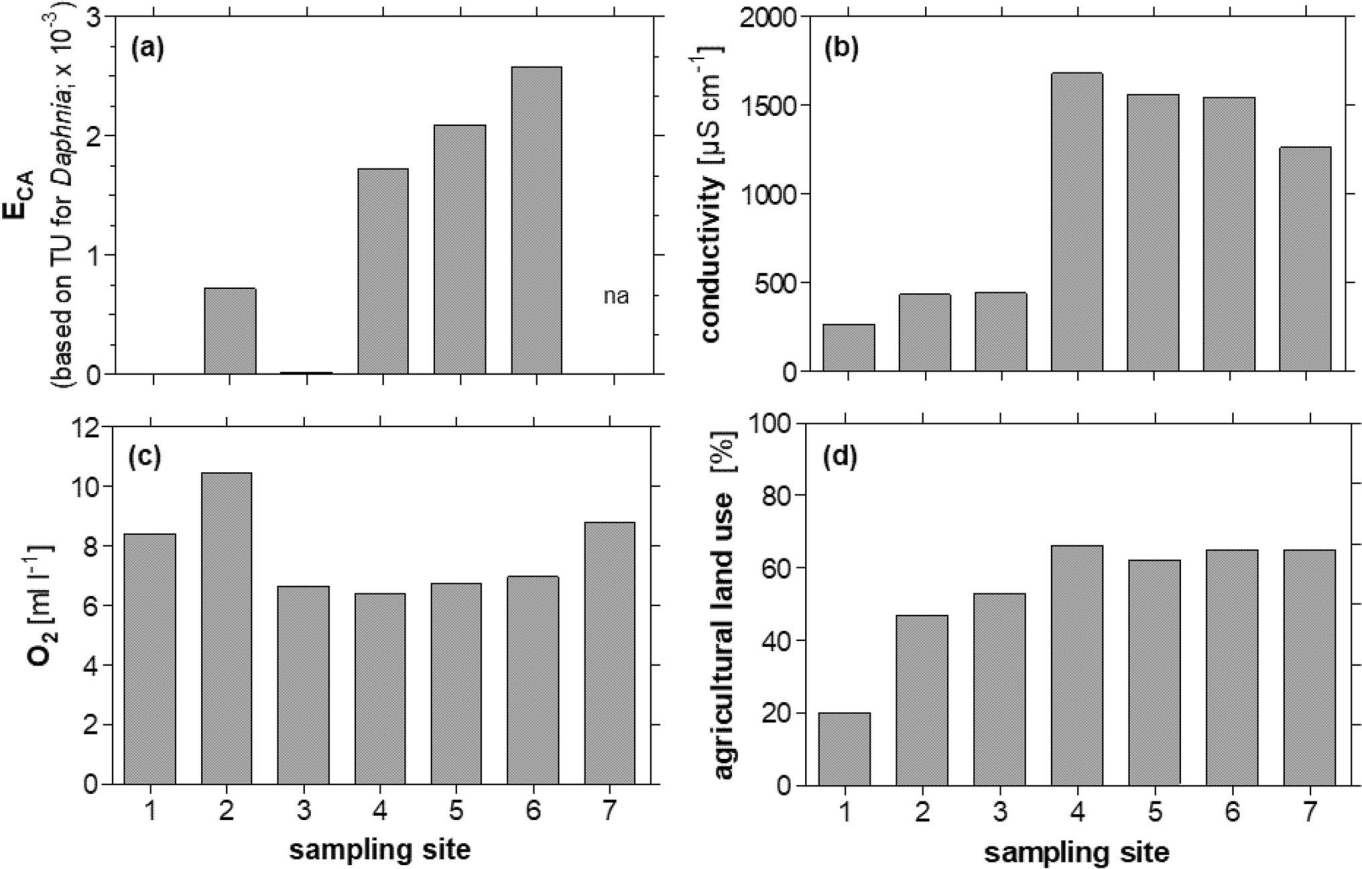
Selected abiotic parameters from most upstream site 1 to downstream sampling site 7. **a** Mixture effect (*E*_CA_) based on the sum of toxic units (TU) in *Daphnia* spp. for 57 analyzed chemicals according to Table S4. **b** Conductivity, **c** oxygen (O_2_) content, and **d** agricultural land use

**Fig. 4:**
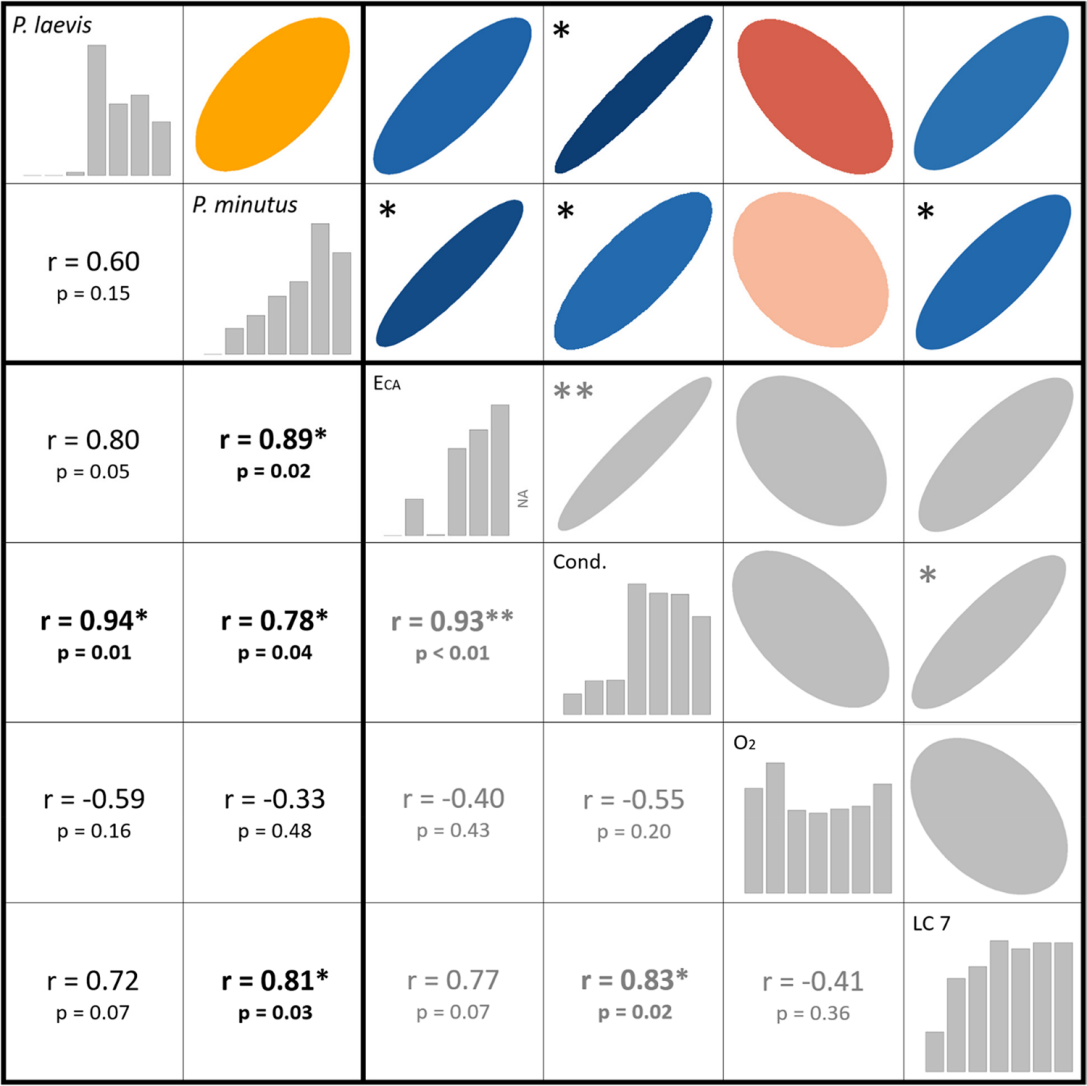
Correlation matrix relating environmental data and Acanthocephala prevalence. Shown are Pearson correlation coefficients. The environmental variables shown are mixture effect (*E*_CA_ based on the sum of toxic units (TU) in *Daphnia* spp. for 57 analyzed chemicals according to Appendix 1, Table S4; [10^−3^]), conductivity (cond.; [µS/cm]), oxygen content (O_2;_ [mg/l]) and agricultural land use (LC7; [%]). Significant (*p* < 0.05, *p* < 0.01) relationships are marked with asterisks

We found a strong variation of infection patterns of *G. roeselii* across sampling sites and acanthocephalan species (*Pomphorhynchus laevis* and *Polymorphus minutus*; Appendix 1: Table S2), related to changing environmental conditions along the river gradient (Table 1). Upstream reaches (site 1–3) of the Horloff were characterized by low prevalence (P) with *P. minutus* (*P* ≤ 2.68%) and *P. laevis* (*P* ≤ 1.79%; Fig. 2). Co-infections of amphipods with both parasite species occurred in 11 individuals. Conductivity increased due to the discharge of first small WWTPs (between site 1 and 3), but this discharge did not affect the mixture effect (*E*_CA_ according to formula 2) as sum of toxic units and the parasite prevalence. Prevalence in the middle reaches (site 4–6) then increased sharply, with *P. laevis* being the dominant species. Highest prevalence (*P* ≤ 73%)—and intensities of up to 9 individuals of *P. laevis* in one amphipod—occurred at site 4 (mI ≤ 3; Fig. 2e), which coincides with an increased *E*_CA_, and a sharp increase in carbonate hardness, conductivity, pH and temperature from sampling site 3 to 4 (Table 1, Fig. 3). The overall correlation of *E*_CA_ and prevalence of *P. laevis* was slightly non-significant (*n* = 6; Pearson’s *r* = 0.80; *p* = 0.05), while prevalence of *P. minutus* significantly increased with higher *E*_CA_ (*n* = 6; Pearson’s *r* = 0.89; *p* = 0.02). Furthermore, the prevalence of both *P. laevis* and *P. minutus* correlated significantly with conductivity (Fig. 4).

**Table 1:** Environmental parameters at the sampling sites. Water parameters (temperature–total hardness were recorded as single measurements during biological sampling), percentage of agriculture area/cultivated vegetation areas across the subcatchment (weighted average) taken from Domisch et al. (2015), mixture effect (*E*_CA_) was calculated on the basis of toxic units for single compounds in the sample (see Material and Methods and Appendix 1: Table S4), CPUE (*n* captured per person per hour) of *Gammarus roeselii* during sampling in September/October 2020

Site ID	River	Temperature [°C]	Conductivity [µS/cm]	pH	O_2_ [mg/l]	O_2_ [%]	Nitrite [mg/l]	Nitrate [mg/l]	Carbonate hardness [°dKH]	Total hardness [°dGH]	Agricultural land use [%]	*E*_CA_ [× 10^−3^]	*G. roeselii* [CPUE]
1	Horloff	15.5	265	7.00	8.41	87.1	0.0	0.0	7.2	7.6	20	4.0 × 10^−5^	45
2	Horloff	14.5	434	7.93	10.46	105.4	0.15	0.0	10.6	11.8	47	0.72	25
3	Horloff	15.8	443	7.00	6.65	69.0	0.025	0.0	11.0	12.2	53	0.019	940
4	Horloff	18.4	1678	7.88	6.41	70.0	0.15	0.0	27.2	15.2	66	1.72	732
5	Horloff	18.8	1560	7.92	6.75	74.4	0.025	0.0	27.2	16.0	62	2.09	418
6	Horloff	17.2	1545	7.49	6.97	74.3	0.0	0.0	27.8	14.8	65	2.58	683
7	Nidda	12.8	1261	7.69	8.77	87.1	0.0	10.0	11.2	14.0	65	na	154

Downstream from sampling site 3, a large WWTP discharges treated wastewater (78,000 person equivalents; Fig. 1). From sampling site 4 on, conductivity remained constant and the *E*_CA_ increased only slightly further along the river to sampling site 6. Parasite prevalence of *P. laevis* between sampling site 5 and 7 remained at a high level, ranging between 30 and 45% with a mean intensity of 1.88. Some co-infections of *G. roeselii* with *P. minutus* and *P. laevis* (Fig. 2b) were recorded from site 4 downstream. Highest prevalence of *P. minutus* was found in amphipods collected at site 6 (*P* ≤ 9%) and one parasite per amphipod host was the maximum intensity recorded from the river Horloff. The correlation of amphipod size (mm) and number of parasites was weak but significant (all amphipods: *n* = 674; Pearson's *r* = 0.174; *p* < 0.001; infected amphipods: *n* = 208; Pearson’s *r* = 0.191, *p* = 0.006).

### Acute toxicity tests

**Fig. 5:**
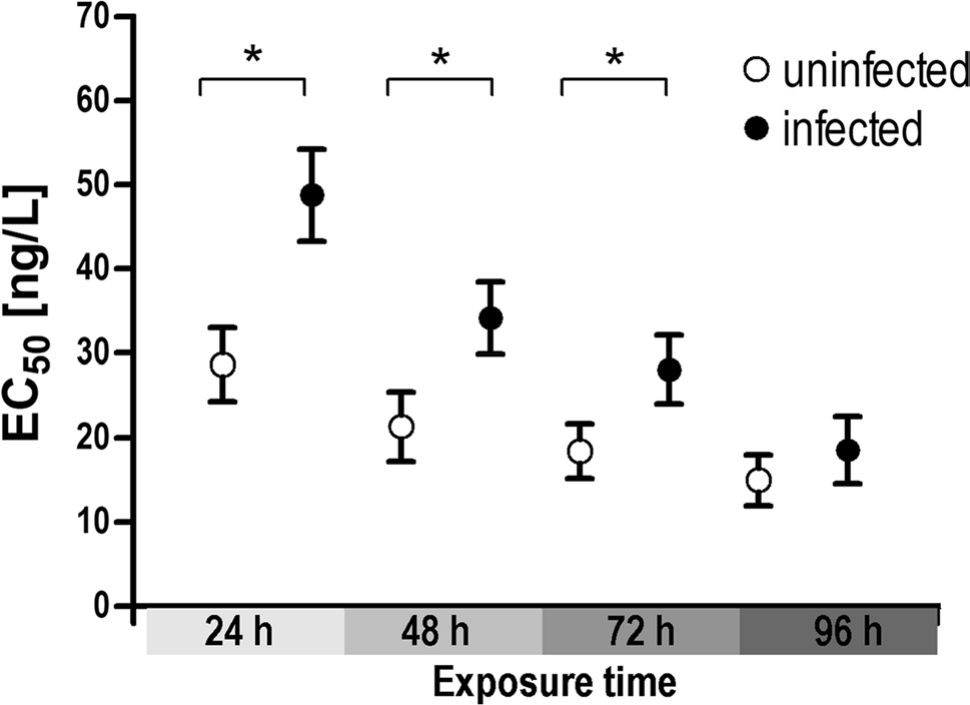
Effect concentrations (EC_50_, mean ± SD) of uninfected and infected *Gammarus roeselii* after 24 h, 48 h, 72 h, and 96 h exposure to deltamethrin. For dose–response curves, see also Appendix 1, Figure S4

The acute toxicity tests have shown clear differences in sensitivity between infected and uninfected *G. roeselii* after exposure to deltamethrin. In general, immobility increased over time (Appendix 1: Figure S4), but showed significant infection-dependent differences after 24, 48, and 72 h (Table 2; Fig. 5). The effect concentration (EC_50_) was 48.7 ng/l for infected *G. roeselii* after 24 h and 28.6 ng/l for the uninfected amphipods (Table 2). The EC_50_ of infected *G. roeselii* was always higher than of uninfected *G. roeselii* at the respective time of exposure, however, the difference decreased with increasing duration of deltamethrin exposure and was no longer significant after 96 h. After 96 h, the EC_50_ value of the infected amphipods was 18.5 ng/l and 14.9 ng/l of uninfected amphipods. Mobility (i.e., survival) in the negative- and solvent controls was always ≥ 90%, therefore meeting the validity criterion for the toxicity test.

**Table 2:** EC_50_ (ng/l) of infected and uninfected *G. roeselii* after 24 h, 48 h, 72 h, and 96 h exposure to deltamethrin. Differences in EC_50_ parameters were computed as ratios of the estimated model parameters (EC_50uninfected_/EC50_50infected_) using *compParm* function in the drc package

Exposure time	Infection status	EC_50_ ± SE	Comparison of EC_50_ values (estimate ± SE)	Comparison of EC_50_ values (*t* value)	Comparison of EC_50_ values (*p* value)
24 h	Uninfected	28.6 ± 4.42	0.587 ± 0.112	− 3.674	< 0.001 ***
	Infected	48.7 ± 5.49			
48 h	Uninfected	21.3 ± 4.10	0.623 ± 0.143	− 2.628	0.009 **
	Infected	34.1 ± 4.27			
72 h	Uninfected	18.4 ± 3.24	0.656 ± 0.150	− 2.305	0.021 *
	Infected	28.1 ± 4.06			
96 h	Uninfected	15.0 ± 3.03	0.808 ± 0.239	− 0.805	0.421
	Infected	18.5 ± 3.97			

## Discussion

We investigated acanthocephalan infection levels of *G. roeselii* along a river gradient that is under strong anthropogenic pressure and further asked whether the infection with acanthocephalans influences the sensitivity of amphipods to anthropogenic stressors such as pesticides. With a prevalence of up to 73% and intensities of up to nine individuals of *P. laevis*, we found an unusually high infection rate of *G. roeselii* in the downstream reaches of our study system. At the same time, infected *G. roeselii* showed a higher tolerance to the pyrethroid insecticide deltamethrin, suggesting that infection with acanthocephalans can even have beneficial effects (Fig. 5).

### Infection patterns across river gradient

Acanthocephalan infection rates of intermediate crustacean hosts are usually described at very low levels, ranging between 0.01 and 1% prevalence (Busch et al. 2012; Emde et al. 2014). However, similar to the present study, a high prevalence of *P. laevis* (acanthella or cystacanth stage) was also found in the amphipod *Echinogammarus stammeri* in Italy, with a prevalence of > 90% and intensities ranging between 1 and 15 during late summer, while prevalence of parasites in amphipods 2 km further downstream were significantly lower over a period of 7 years (Dezfuli et al. 1999). The authors explained this difference with varying abundances of preferred final hosts and paratenic hosts, which might—at least to some extent—explain the low *P. laevis* prevalence at upstream sites in our study. Approximately 80% of the fish fauna further upstream of the Horloff consists of *Phoxinus phoxinus, Barbatula barbatula, Gobio gobio*, and *Leuciscus leuciscus*, species likely functioning as paratenic hosts for *P. laevis* (Médoc et al. 2011; Perrot-Minnot et al. 2020). Paratenic hosts are not considered to be obligatory, but very often, they bridge a trophic gap between intermediate and final hosts, but without further development (Schmidt 1985). A minimum of 50% of the fish fauna further downstream is composed of paratenic hosts as well, e.g., *Rutilus rutilus* and *Gobio gobio*, but includes at the same time a larger proportion of species functioning as final hosts, e.g., *Barbus barbus, Perca fluviatilis* or *Squalius cephalus* (Perrot-Minnot et al. 2020). The latter species has recently been identified as a main final host for *P. laevis* (Perrot-Minnot et al. 2019). We do not have specific information on the occurrence of birds, e.g. ducks and great crested grebes, which function as final hosts of *P. minutus*. However, we assume that there are more species of waterfowl (e.g., swans and ducks) on the banks of the lower, deeper reaches (Cramp 1977) than on the upper, very shallow reaches (< 25 cm water depth) surrounded by forest that might to some extent explain the steady increase in prevalence towards downstream reaches (for pictures of sampling sites see also Appendix 1, Fig. S2).

The high prevalence of *P. laevis* in *G. roeselii* at downstream sites could also be facilitated by the lack of behavioral manipulation due to the parasites and thus a non-increased (i.e., normal) predation risk: It is known that *P. laevis* can manipulate the phototactic behavior of closely related *Gammarus pulex*—a behavior that aims to reduce the risk of predation—but fails to manipulate behavior of *G. roeselii* (Bauer et al. 2000). The shorter co-evolutionary history between *P. laevis* and *G. roeselii* may explain the lack of adaptation of the parasite to the host (Moret et al. 2007). More than that, it even seems that through a substantial immune response *G. roeselii* is even better adapted to the parasite than vice versa (Rigaud and Moret 2003; Moret et al. 2007). However, the actual contribution of altered photophobia to parasite transmission has been questioned, suggesting that altered photophobia is not the main causative agent of the increased vulnerability of infected amphipods to predation by fish (Perrot-Minnot et al. 2012). The quantification of behavioral manipulation—especially under exposure of chemical pollutants—is therefore a promising future field of research in this system (Ford et al. 2021).

Another explanation for the unusually high prevalence is provided by the second part of our study and refers to the higher tolerance of infected amphipods to chemical stressors in general and the pesticide deltamethrin in particular, suggesting a beneficial effect of acanthocephalan infection for *G. roeselii* at polluted sites further downstream. The marked increase in prevalence of *P. laevis* at sampling site 4 is linked with the discharge of treated wastewater from a large WWTP upstream from this location and the associated increase in the mixture effect *E*_CA_. Even though the overall correlation of *E*_CA_ and prevalence of *P. laevis* was slightly non-significant, the increase of *E*_CA_ and prevalence of *P. laevis* at site 4 is striking. The time gap between chemical monitoring (i.e., the calculation of mixture toxicity *E*_CA_) and biological monitoring remains a limitation of our study. Nevertheless, we expect a similar persistent anthropogenic pressure on our study system, as agricultural practices, technical equipment, and operation procedures of the WWTPs have not changed during the time of our study. The strong correlation of conductivity and *E*_CA_ also indicates that the sum of substances introduced was similar at both time periods.

### Increased tolerance of infected individuals

Our study was premised on the hypothesis that the infection with acanthocephalans influences the sensitivity of amphipods to a specific chemical stressor. To test this hypothesis, we analyzed the responses of uninfected and infected *G. roeselii* to the commonly used insecticide deltamethrin under controlled conditions in the laboratory. Our acute toxicity tests indicated higher tolerance of individuals with acanthocephalan infections, especially in the case of short-term exposure (i.e., during the first 72 h). Such a short-term pulse load is a common scenario in agriculturally dominated river systems after heavy rain with resulting run-off events from agricultural sites (Weston and Lydy 2010), and infected amphipods appear to have an increased probability of survival compared to uninfected individuals. With longer exposure, the effect continuously disappears and both infected and uninfected individuals are subject to equally high mortality from deltamethrin. Long-term exposure with insecticides might occur at sites where deltamethrin accumulates in the sediment, thereby exerting stress onto bottom-dwelling amphipods. A study by Tucca et al. (2014) reported an LC_50_ of 7.8 µg/kg in a whole sediment testing for a marine amphipod after 10 days of deltamethrin exposure, which suggests similar toxicities of deltamethrin after acute and long-term exposure in water and sediment, respectively. Further evidence why infection of acanthocephalans may be beneficial in amphipods comes from a recent study: *G. fossarum* infected with *P. minutus* had significantly higher lipid and glycogen content than uninfected *G. fossarum* (Rothe et al. 2022), which may also have positively affected the resistance of infected organisms to deltamethrin exposure in our test.

Deltamethrin is a highly lipophilic substance (log *K*_OW_ = 5.4, Kidd and James 1991). Due to its lipophilicity, deltamethrin partitions from the water into suspended solids, sediments and biota (Pawlisz et al. 1998) and is therefore readily taken up by amphipods. Thus, the insecticide should come into direct contact with the larval stages of acanthocephalans via the parasite’s tegument once it has entered the hemocoel of the amphipod. Different studies have shown that adult acanthocephalans are able to accumulate more toxic chemicals (e.g., heavy metals and organic chemicals such as pentachlorophenol) than their hosts, and increased heavy metal concentrations in the parasite are often accompanied by decreased toxicant concentrations in the host compared to non-infected conspecifics (see review Sures et al. 2017). On the other hand, accumulation of pollutants (lead or cadmium) in cystacanth stages of *P. laevis* was lower compared to the intermediate host tissue of *G. pulex* (Brown and Pascoe 1989; Siddall and Sures 1998) and also in *P. minutus* compared to the host tissue of *G. roeselii* (Gismondi et al. 2012), although higher palladium levels were measured in cystacanths of *P. minutus* than in tissue of *G. roeselii* (Sures and Radszuweit 2007). Overall, these results suggest that effects of parasites might vary depending on the host-parasite system as well as the chemical stressor and future analytical studies are needed to determine the actual pollutant load in host tissue and parasite tissue.

Deltamethrin is one of the most toxic pyrethroid insecticides for crustaceans, possibly due to their low capacity to detoxify (Pérez-Fernández et al. 2010). High levels of lipid peroxidation (Oliveira et al. 2012) and short-term oxidative damages (Dorts et al. 2009) have been observed in marine crustaceans, possibly linked to the failing of the antioxidant protection by glutathione S-transferase enzymes with increasing deltamethrin concentrations. Acanthocephalans appear to alleviate some of the negative effects of deltamethrin, but further studies are needed to improve the mechanistic understanding of parasite effects on the stress response and detoxification mechanisms of the amphipod hosts (Grabner and Sures 2019). In general, *G. roeselii*, like other gammarids, is very sensitive to deltamethirn. Lethal limits were reported for *G. fossarum* and *G. pulex*, with a 96-h LC_50_ of 33.2 and 68.0 ng/l, respectively (Adam et al. 2010). However, some variability in the response to deltamethrin was reported for populations of *Gammarus* sp., suggesting potential adaptations to the stressor in some populations (Adam et al. 2010; Grethlein et al. 2022). Our study complements these findings and shows that there may be differences in sensitivity not only between populations of the same species, but even within a population, depending on acanthocephalan infections.

## Conclusion and outlook

While reliable cause-and-effect studies are challenging, the combined effects of parasites in intermediate and final hosts and pollutants are increasingly studied as part of the recently established research direction ‘Environmental Parasitology’ (Giari et al. 2020). Whether negative effects of parasite infections, e.g., the reduction of growth, respiration rate, or egg production of acanthocephalan species on intermediate hosts (Kennedy 2006) are moderated by the potential positive effects of reduced contaminant exposure requires further investigation.

While we chose to study the effects of deltamethrin in water, other ubiquitous pesticides should be tested and further include sediment bioassays to better mimic the setting in the field. Accumulation of deltamethrin in individual parasites and the infected and uninfected amphipod host needs to be quantified to confirm the positive “buffer” effect of acanthocephalans, i.e. an increase in the survival rate of the host in an exposure scenario. Regular environmental monitoring activities might benefit from additional variables such as parasite prevalence in intermediate hosts, but also in fish hosts, to detect potential patterns related to seasonal pesticide exposure.

## Supplementary Information

Below is the link to the electronic supplementary material.

Supplementary file1 (PDF 1576 KB)

## Data Availability

Raw data are included in the supplementary material.
