# Supplementary material for: Infection with acanthocephalans increases tolerance of Gammarus roeselii (Crustacea: Amphipoda) to pyrethroid insecticide deltamethrin
Source: Environ Sci Pollut Res Int. 2023 Mar 10;30(19):55582–95. doi: 10.1007/s11356-023-26193-0 (PMC10121498; doi:10.1007/s11356-023-26193-0)
Supplement: Supplementary file 1 — Supplementary file1 (PDF 1576 KB) [file 11356_2023_26193_MOESM1_ESM.pdf]

# Supplementary Material

## Infection with acanthocephalans increases tolerance of *Gammarus roeselii* (Crustacea: Amphipoda) to pyrethroid insecticide deltamethrin

Judith Kochmann[1,2*], Melanie Laier[1,4], Sven Klimpel[1,2], Arne Wick[3], Uwe Kunkel[3,‡], Jörg Oehlmann[4] and Jonas Jourdan[4*]

## Appendix A – Additional parameters

**Table S1** Geographical coordinates of amphipod sampling sites (this study, sampling in 2020), sampling sites for chemical analyses (this study, sampling in 2015 and 2016), fish sampling sites (sampling between 2007-2018 by the Hessian Agency for Nature Conservation, Environment and Geology), and wastewater treatment plants.

| **Amphipod sampling site** | **Sampling site for chemical analyses** | N | E |
|---|---|---|---|
| **1** | **I** | 50°31'08.68" | 09°02'24.19" |
| | **Ia** | 50°31'15.0" | 08°58'56.0" |
| **2** | **II** | 50°30'54.99" | 08°57'27.76" |
| **3** | | 50°28'17.14" | 08°54'03.96" |
| | **III** | 50°26'14.1" | 08°53'54.1" |
| | **IIIa** | 50°26'07.6" | 08°53'48.3" |
| **4** | **IV** | 50°24'36.67" | 08°54'03.03" |
| | **IVa** | 50°23'57.0" | 08°53'56.0" |
| | **IVb** | 50°23'38.6" | 08°53'44.1" |
| | **IVc** | 50°23'14.8" | 08°53'26.0" |
| **5** | **V** | 50°22'48.01" | 08°53'16.43" |
| | **Va** | 50°21'38.6" | 08°52'43.4" |
| | **Vb** | 50°20'43.8" | 08°52'34.1" |
| **6** | **VI** | 50°19'44.26" | 08°52'13.80" |
| **7** | | 50°13'23.2" | 08°46'08.2" |

| | | |
|---|---|---|
| **Fish sampling site** | N | E |
| **a** | 50°24'41.32" | 08°54'04.83" |
| **b** | 50°23'45.94" | 08°53'48.84" |
| **c** | 50°25'04.27" | 08°54'00.93" |
| **d** | 50°20'39.18" | 08°52'33.70" |
| **e** | 50°30'49.90" | 08°56'55.02" |
| **f** | 50°13'18.53" | 08°46'10.00" |
| | | |
| **Wastewater treatment plants (person equivalents)** | N | E |
| Gonterskirchen (990) | 50°30'44.11" | 09°01'01.84" |
| Friedrichshütte (100) | 50°31'14.45" | 08°58'58.60" |
| Ruppertsburg (990) | 50°30'51.92" | 08°57'01.18" |
| Hungen-Utphe (78,000) | 50°26'12.45" | 08°53'52.32" |
| Nidda (35,000) | 50°23'55.69" | 08°59'15.98" |
| Dauernheim (7,500) | 50°21'27.44" | 08°57'14.26" |
| Florstadt (30,000) | 50°18'31.45" | 08°50'40.64" |
| Assenheim (7,000) | 50°17'29.08" | 08°48'40.26" |
| Nieder-Wöllstadt (12,000) | 50°16'19.28" | 08°46'53.64" |
| Karben (40.000) | 50°13'47.96" | 08°45'50.33" |

**Table S2** Parasitological parameters of *Gammarus roeselii* from seven different sampling stations.

| **Site** | **P [%]** | | **$I_{min}$-$I_{max}$** | | **mI** | | **mA** | |
|---|---|---|---|---|---|---|---|---|
| | *P. laevis* | *P. minutus* | *P. laevis* | *P. minutus* | *P. laevis* | *P. minutus* | *P. laevis* | *P. minutus* |
| **1** | 0 | 0 | 0 | 0 | 0 | 0 | 0 | 0 |
| **2** | 0 | 1.79 | 0 | 1 | 0 | 1 | 0 | 0.02 |
| **3** | 1.79 | 2.68 | 1 | 1 | 1 | 1 | 0.02 | 0.03 |
| **4** | 73 | 4 | 1 – 9 | 1 | 3 | 1 | 2.19 | 0.04 |
| **5** | 40 | 5 | 1 – 7 | 1 | 1.88 | 1 | 0.75 | 0.05 |
| **6** | 45 | 9 | 1 – 5 | 1 | 1.44 | 1 | 0.65 | 0.09 |
| **7** | 30 | 7 | 1 – 4 | 1 – 2 | 1.37 | 1.14 | 0.41 | 0.08 |

I = Intensity, mA = mean abundance, mI = mean intensity and P = prevalence

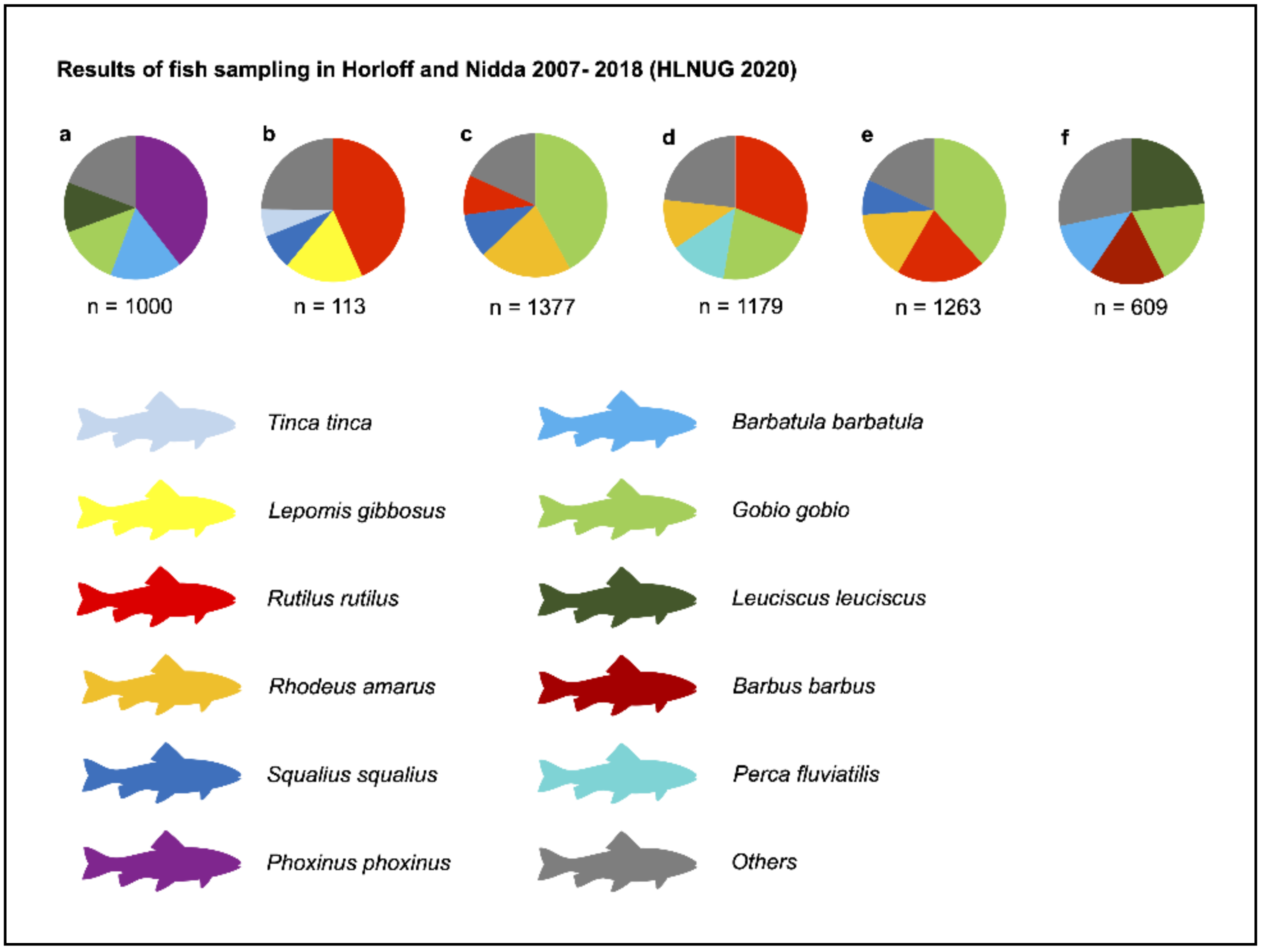


**Figure S1** Results of fish sampling in Horloff and Nidda at locations a-f between 2007-2018 by the Hessian Agency for Nature Conservation, Environment and Geology. For exact locations, refer to Figure 1 in the main manuscript and Table S1; n = total number of individuals.

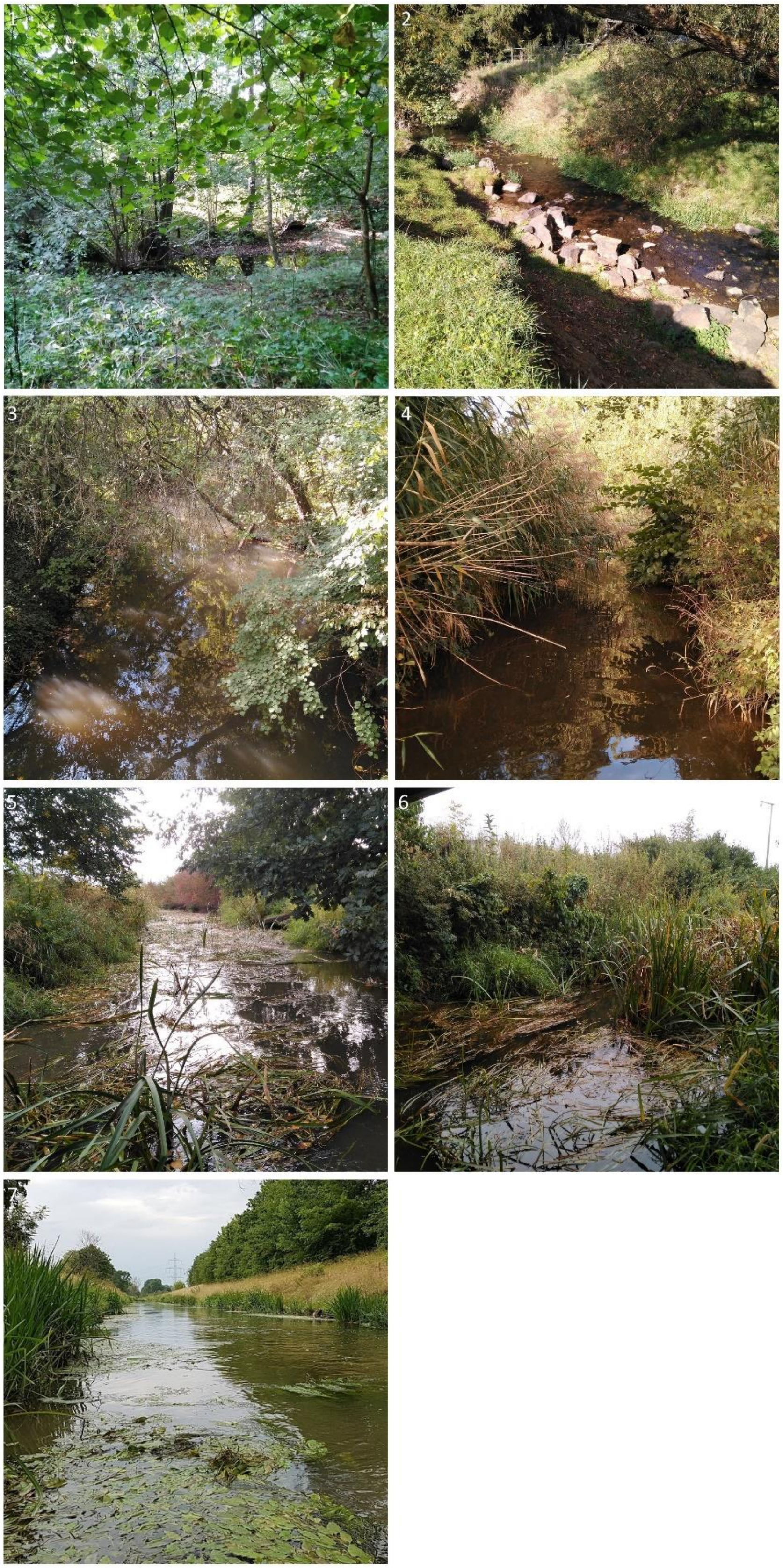


**Figure S2** photos from sampling site 1 to 7.

**Table S3** List of analysed substances with mean measured concentrations between July 2015 and July 2016.

| Chemical | CAS_NO | LOQ (ng/l) | mean measured concentration (ng/L) at sampling site | | | | | | | | | | | | |
|---|---|---|---|---|---|---|---|---|---|---|---|---|---|---|---|
| | | | I | Ia | II | III | IIIa | IV | IVa | IVb | ÎVc | V | Va | Vb | VI |
| **Pharmaceuticals** | | | | | | | | | | | | | | | |
| Acetaminophen | 103-90-2 | 10 | <LOQ | <LOQ | <LOQ | <LOQ | <LOQ | <LOQ | <LOQ | <LOQ | <LOQ | <LOQ | <LOQ | <LOQ | <LOQ |
| Aciclovir | 59277-89-3 | 20 | <LOQ | <LOQ | <LOQ | <LOQ | <LOQ | <LOQ | <LOQ | <LOQ | <LOQ | <LOQ | <LOQ | <LOQ | <LOQ |
| Aliskiren | 173334-57-1 | 10 | <LOQ | <LOQ | 16.5 | <LOQ | 82.9 | 58.8 | 61.8 | 17.7 | 61.7 | 54.4 | 29.0 | 22.6 | 30.5 |
| Amisulpride | 71675-85-9 | 5 | <LOQ | <LOQ | <LOQ | <LOQ | 62.3 | 32.7 | 28.2 | 10.6 | 41.7 | 32.9 | 17.5 | 16.8 | 17.8 |
| Atenolol | 29122-68-7 | 10 | <LOQ | <LOQ | <LOQ | <LOQ | <LOQ | <LOQ | <LOQ | <LOQ | <LOQ | <LOQ | <LOQ | <LOQ | <LOQ |
| Azithromycin | 83905-01-5 | 20 | <LOQ | <LOQ | <LOQ | <LOQ | 32.1 | <LOQ | <LOQ | <LOQ | <LOQ | <LOQ | <LOQ | <LOQ | <LOQ |
| Bezafibrate | 41859-67-0 | 5 | <LOQ | <LOQ | 18.1 | 5.1 | 12.8 | 17.0 | 20.1 | 16.3 | <LOQ | <LOQ | 5.8 | 5.8 | 8.1 |
| Bicalutamide | 90357-06-5 | 2 | <LOQ | <LOQ | 2.4 | <LOQ | 4.5 | 3.9 | 4.6 | 2.7 | 4.7 | 5.1 | 2.3 | 2.5 | 2.9 |
| Candesartan | 139481-59-7 | 2 | <LOQ | 14.1 | 94.5 | 32.0 | 394.8 | 351.3 | 334.7 | 310.8 | 365.5 | 345.4 | 160.6 | 183.9 | 211.2 |
| Carbamazepine | 298-46-4 | 1 | <LOQ | <LOQ | 50.4 | 19.5 | 223.5 | 189.9 | 186.2 | 184.7 | 168.6 | 156.7 | 66.0 | 75.0 | 129.3 |
| Cetirizine | 83881-51-0 | 5 | <LOQ | <LOQ | 14.8 | <LOQ | 51.8 | 39.5 | 41.0 | 30.2 | 50.0 | 42.3 | 20.5 | 22.6 | 23.1 |
| Chlorothiazide | 58-94-6 | 2 | <LOQ | 8.4 | 19.8 | 16.8 | 106.0 | 83.7 | 77.3 | 52.8 | 80.6 | 67.6 | 63.2 | 58.9 | 50.6 |
| Citalopram | 59729-33-8 | 5 | <LOQ | <LOQ | 6.0 | <LOQ | 36.8 | 11.8 | 7.7 | 7.4 | 7.2 | 5.4 | 5.2 | 5.1 | 7.2 |
| Clarithromycin | 81103-11-9 | 2 | <LOQ | <LOQ | 13.0 | <LOQ | 10.1 | 12.2 | 10.3 | 3.8 | 4.6 | 3.8 | 3.0 | 3.1 | 2.4 |
| Clopidogrel | 90055-48-4 | 2 | <LOQ | <LOQ | <LOQ | <LOQ | 2.3 | <LOQ | <LOQ | <LOQ | <LOQ | <LOQ | <LOQ | <LOQ | <LOQ |
| Codeine | 76-57-3 | 10 | <LOQ | <LOQ | <LOQ | <LOQ | <LOQ | <LOQ | <LOQ | <LOQ | <LOQ | <LOQ | <LOQ | <LOQ | <LOQ |
| Diclofenac | 15307-86-5 | 2 | <LOQ | <LOQ | 138.9 | 47.6 | 536.7 | 426.4 | 356.4 | 355.3 | 224.6 | 236.3 | 233.4 | 239.7 | 187.0 |
| Diphenhydramine | 58-73-1 | 1 | <LOQ | <LOQ | 1.4 | 1.1 | 12.9 | 3.5 | 2.3 | 2.2 | 1.5 | 1.4 | 1.8 | 1.8 | 1.1 |
| Dipyridamole | 58-32-2 | 10 | <LOQ | <LOQ | <LOQ | <LOQ | <LOQ | <LOQ | <LOQ | <LOQ | <LOQ | <LOQ | <LOQ | <LOQ | <LOQ |
| Emtricitabine | 143491-57-0 | 5 | <LOQ | <LOQ | <LOQ | <LOQ | 5.3 | <LOQ | <LOQ | <LOQ | <LOQ | <LOQ | <LOQ | <LOQ | 4.5 |
| Enalaprilat | 76420-72-9 | 20 | <LOQ | <LOQ | <LOQ | <LOQ | <LOQ | <LOQ | <LOQ | <LOQ | <LOQ | <LOQ | <LOQ | <LOQ | <LOQ |
| Erythromycin | 114-07-8 | 5 | <LOQ | <LOQ | <LOQ | <LOQ | 9.0 | 6.1 | 5.6 | 7.1 | 9.6 | 6.6 | 5.8 | 5.6 | <LOQ |
| Fexofenadine | 83799-24-0 | 2 | <LOQ | <LOQ | 37.7 | <LOQ | 87.5 | 61.9 | 103.9 | 40.8 | 51.9 | 49.0 | 30.9 | 34.3 | 30.8 |
| Flecainide | 54143-55-4 | 2 | <LOQ | 4.5 | 26.6 | 7.3 | 93.0 | 72.3 | 70.0 | 31.3 | 63.0 | 54.0 | 42.4 | 35.5 | 40.9 |
| Fluconazol | 86386-73-4 | 5 | <LOQ | <LOQ | 66.2 | 41.5 | 28.9 | 23.4 | 22.1 | 18.4 | 27.4 | 24.5 | 16.5 | 17.6 | 16.7 |
| Furosemide | 54-31-9 | 10 | <LOQ | <LOQ | 19.5 | <LOQ | 65.3 | 46.9 | 47.2 | 26.3 | 21.2 | 19.4 | 19.1 | 18.6 | 18.3 |
| Gabapentin | 60142-96-3 | 20 | <LOQ | 135.8 | 458.2 | 313.6 | 540.9 | 549.3 | 712.4 | 262.9 | 416.5 | 435.8 | 224.0 | 310.9 | 379.1 |
| Hydrochlorothiazide | 58-93-5 | 10 | <LOQ | 124.5 | 382.7 | 195.0 | 1669.8 | 1290.1 | 1171.7 | 689.5 | 980.0 | 802.3 | 708.4 | 634.9 | 584.5 |
| Ibuprofen | 15687-27-1 | 20 | <LOQ | <LOQ | <LOQ | <LOQ | <LOQ | <LOQ | <LOQ | <LOQ | <LOQ | <LOQ | <LOQ | <LOQ | 46.3 |
| Irbesartan | 138402-11-6 | 10 | <LOQ | 38.3 | 112.8 | 67.6 | 597.9 | 491.1 | 459.1 | 238.9 | 422.6 | 357.7 | 173.1 | 186.3 | 277.6 |
| Lamotrigine | 84057-84-1 | 20 | <LOQ | <LOQ | 25.8 | <LOQ | 171.3 | 126.2 | 135.3 | 98.9 | 149.9 | 140.9 | 148.0 | 104.8 | 83.5 |
| Levetiracetam | 102767-28-2 | 50 | <LOQ | <LOQ | <LOQ | <LOQ | <LOQ | <LOQ | <LOQ | <LOQ | <LOQ | <LOQ | <LOQ | <LOQ | 50.8 |
| Lidocaine | 137-58-6 | 2 | <LOQ | 4.4 | 17.6 | 7.0 | 37.2 | 35.4 | 33.3 | 25.9 | 24.8 | 21.4 | 21.7 | 22.7 | 18.5 |
| Metoprolol | 37350-58-6 | 5 | <LOQ | 16.2 | 43.4 | 14.9 | 141.8 | 107.7 | 115.8 | 77.4 | 71.3 | 61.2 | 54.6 | 49.2 | 63.5 |
| Naproxen | 22204-53-1 | 20 | <LOQ | <LOQ | 22.1 | <LOQ | <LOQ | 23.0 | 40.0 | 22.6 | <LOQ | <LOQ | <LOQ | <LOQ | <LOQ |
| Olmesartan | 144689-24-7 | 20 | <LOQ | <LOQ | 40.9 | <LOQ | 127.4 | 125.1 | 130.4 | 49.4 | 119.1 | 101.9 | 36.3 | 40.1 | 68.3 |
| Oxazepam | 604-75-1 | 5 | <LOQ | 15.8 | 10.9 | 13.0 | 58.4 | 45.5 | 37.9 | 36.1 | 41.8 | 34.8 | 31.2 | 29.4 | 38.3 |
| Oxcarbazepine | 28721-07-5 | 2 | <LOQ | <LOQ | <LOQ | <LOQ | <LOQ | 30.0 | 19.0 | 17.8 | 12.3 | 9.1 | 6.2 | 4.1 | 3.0 |
| Phenytoin | 57-41-0 | 5 | <LOQ | <LOQ | <LOQ | <LOQ | 7.8 | 10.0 | 8.4 | <LOQ | 7.0 | 5.9 | <LOQ | <LOQ | <LOQ |
| Pregabalin | 148553-50-8 | 10 | <LOQ | <LOQ | 38.2 | <LOQ | 52.9 | 71.9 | 202.9 | 28.2 | 36.7 | 39.1 | 23.4 | 27.2 | 26.7 |
| Primidone | 125-33-7 | 20 | <LOQ | <LOQ | <LOQ | <LOQ | 135.0 | 128.4 | 123.7 | 51.6 | 100.6 | 93.2 | 89.8 | 75.1 | 83.0 |
| Ramipril | 87333-19-5 | 20 | <LOQ | <LOQ | <LOQ | <LOQ | <LOQ | <LOQ | <LOQ | <LOQ | <LOQ | <LOQ | <LOQ | <LOQ | <LOQ |
| Ranitidine | 66357-35-5 | 10 | <LOQ | <LOQ | <LOQ | <LOQ | 32.3 | 17.9 | <LOQ | <LOQ | <LOQ | <LOQ | <LOQ | <LOQ | <LOQ |
| Sitagliptin | 486460-32-6 | 5 | <LOQ | 19.0 | 99.1 | 16.7 | 426.2 | 287.6 | 271.7 | 107.5 | 173.9 | 149.1 | 86.5 | 92.2 | 122.3 |
| Sotalol | 3930-20-9 | 10 | <LOQ | <LOQ | 14.4 | <LOQ | 44.7 | 38.2 | 36.4 | 29.6 | 31.1 | 26.5 | 28.8 | 29.0 | 26.1 |
| Sulfamethoxazole | 723-46-6 | 2 | <LOQ | <LOQ | 16.5 | 4.7 | 89.7 | 55.8 | 58.3 | 64.3 | 124.7 | 107.9 | 65.9 | 66.5 | 51.6 |
| Sulpiride | 15676-16-1 | 5 | <LOQ | <LOQ | <LOQ | <LOQ | 74.8 | 50.9 | 43.1 | 47.9 | 52.9 | 43.5 | 40.5 | 31.4 | 34.5 |
| Telmisartan | 144701-48-4 | 5 | <LOQ | 9.7 | 33.9 | 13.8 | 280.0 | 214.7 | 197.2 | 205.9 | 223.1 | 189.9 | 182.2 | 186.5 | 130.5 |
| Torasemide | 56211-40-6 | 2 | <LOQ | 6.3 | 20.7 | 12.8 | 71.7 | 58.9 | 58.0 | 28.7 | 60.0 | 51.9 | 20.4 | 24.5 | 34.9 |
| Tramadol | 27203-92-5 | 1 | 4.5 | 6.0 | 27.0 | 11.5 | 190.2 | 142.3 | 134.5 | 120.3 | 111.0 | 96.7 | 87.7 | 103.0 | 94.9 |
| Trimethoprim | 738-70-5 | 5 | <LOQ | <LOQ | 8.4 | 12.5 | 15.2 | 8.8 | 11.7 | 9.9 | 8.7 | 7.2 | 6.8 | 6.2 | 5.2 |
| Valsartan | 137862-53-4 | 5 | <LOQ | 7.5 | 78.5 | 44.2 | 42.2 | 80.1 | 286.6 | 28.7 | 25.0 | 39.9 | 28.9 | 31.0 | 84.0 |
| Venlafaxine | 93413-69-5 | 2 | <LOQ | 3.6 | 11.4 | 4.1 | 106.7 | 66.2 | 54.5 | 53.7 | 59.8 | 49.6 | 41.8 | 39.3 | 33.8 |
| Xipamide | 14293-44-8 | 2 | <LOQ | <LOQ | 15.5 | <LOQ | 36.2 | 27.0 | 17.7 | 13.7 | 9.8 | 7.1 | <LOQ | <LOQ | <LOQ |
| **Transformation products (TPs) of pharmaceuticals** | | | | | | | | | | | | | | | |
| 5-(3,4,5-Trimethoxybenzoyl)-2,4-pyrimidinediamine | 30806-86-1 | 5 | <LOQ | <LOQ | <LOQ | <LOQ | <LOQ | <LOQ | <LOQ | <LOQ | <LOQ | <LOQ | <LOQ | <LOQ | <LOQ |
| Acetyl-sulfamethoxazole | 21312-10-7 | 5 | <LOQ | <LOQ | 7.3 | <LOQ | 26.6 | 22.0 | 21.9 | 21.4 | 15.6 | 13.5 | 13.7 | 13.1 | 28.1 |
| Acridine-9-carboxylic acid | 5336-90-3 | 5 | <LOQ | <LOQ | 52.8 | 10.3 | 178.8 | 310.8 | 273.6 | 88.3 | 144.5 | 137.7 | 94.4 | 84.0 | 107.4 |
| Acridone | 578-95-0 | 2 | <LOQ | <LOQ | <LOQ | <LOQ | 2.7 | 2.7 | 2.3 | 2.1 | 3.6 | 3.5 | 3.1 | 2.7 | 2.3 |
| Carboxy-aciclovir | 80685-22-9 | 10 | <LOQ | <LOQ | 134.9 | 30.5 | 35.7 | 108.0 | 162.5 | 35.3 | 27.1 | 42.9 | 36.5 | 40.0 | 39.1 |
| O-Desmethyl-amisulpride | 148516-54-5 | 5 | <LOQ | <LOQ | <LOQ | <LOQ | <LOQ | <LOQ | <LOQ | <LOQ | <LOQ | <LOQ | <LOQ | <LOQ | <LOQ |
| Atenolol acid | 56392-14-4 | 10 | <LOQ | 11.8 | 46.3 | 17.8 | 153.1 | 115.4 | 136.7 | 80.2 | 73.3 | 65.0 | 55.3 | 55.1 | 67.4 |
| Hydroxy-atenolol | 68373-10-4 | 10 | <LOQ | <LOQ | <LOQ | <LOQ | <LOQ | <LOQ | <LOQ | <LOQ | <LOQ | <LOQ | <LOQ | <LOQ | <LOQ |
| Dihydro-dihydroxy-carbamazepine | 35079-97-1 | 2 | <LOQ | <LOQ | 67.0 | 24.5 | 363.2 | 329.8 | 340.8 | 142.9 | 242.9 | 237.6 | 118.5 | 134.4 | 227.4 |
| 2-Hydroxy-carbamazepine | 68011-66-5 | 2 | 5.3 | <LOQ | 6.0 | 2.6 | 20.0 | 13.9 | 14.0 | 13.8 | 10.0 | 9.5 | 9.2 | 9.8 | 15.0 |
| 3-Hydroxy-carbamazepine | 68011-67-6 | 2 | <LOQ | <LOQ | 6.4 | <LOQ | 28.6 | 19.9 | 22.3 | 18.1 | 14.4 | 13.4 | 10.2 | 5.9 | 8.0 |
| 10-Hydroxy-carbamazepine | 29331-92-8 | 10 | <LOQ | <LOQ | 17.4 | <LOQ | 136.3 | 109.6 | 115.9 | 81.8 | 76.5 | 71.2 | 70.1 | 72.4 | 73.0 |
| Citalopram-N-oxide | 63284-72-0 | 1 | <LOQ | <LOQ | <LOQ | <LOQ | 2.6 | <LOQ | <LOQ | <LOQ | 1.9 | 1.7 | <LOQ | <LOQ | <LOQ |
| Didesmethyl-citalopram | 166037-78-1 | 10 | <LOQ | <LOQ | <LOQ | <LOQ | <LOQ | <LOQ | <LOQ | <LOQ | <LOQ | <LOQ | <LOQ | <LOQ | <LOQ |
| N-Desmethyl-citalopram | 144010-85-5 | 5 | <LOQ | <LOQ | <LOQ | <LOQ | 24.0 | 10.0 | 7.8 | <LOQ | 7.1 | 5.7 | <LOQ | <LOQ | <LOQ |
| Chloraminophenamide | 121-30-2 | 20 | <LOQ | <LOQ | 77.5 | 27.5 | 394.7 | 279.3 | 254.5 | 278.4 | 432.7 | 344.5 | 261.4 | 194.2 | 150.1 |
| Clopidogrel acid | 144457-28-3 | 5 | <LOQ | 8.6 | 23.5 | 22.1 | 62.0 | 47.8 | 51.6 | 27.7 | 38.6 | 33.4 | 27.2 | 22.5 | 29.3 |
| Carboxy-diclofenac | 13625-57-5 | 2 | <LOQ | <LOQ | 3.0 | 2.5 | 14.8 | 13.2 | 13.1 | 7.9 | 14.4 | 12.5 | 4.9 | 5.5 | 9.2 |
| Diclofenac-lactam | 15362-40-0 | 1 | <LOQ | <LOQ | 1.2 | 1.6 | 15.7 | 10.5 | 9.5 | 7.5 | 10.0 | 8.7 | 4.8 | 5.3 | 6.4 |

| | | | | | | | | | | | | | | | |
|---|---|---|---|---|---|---|---|---|---|---|---|---|---|---|---|
| 4'-Hydroxy-diclofenac | 64118-84-9 | 5 | <LOQ | 6.8 | 17.7 | 8.4 | 99.6 | 67.7 | 25.8 | 24.2 | 7.4 | 6.9 | 7.2 | 7.0 | 13.9 |
| Diphenhydramine-N-oxide | 3922-74-5 | 1 | <LOQ | <LOQ | <LOQ | <LOQ | 4.1 | 1.3 | 1.1 | 1.1 | 3.1 | 2.2 | <LOQ | <LOQ | <LOQ |
| N-Desmethyl-diphenhydramine | 53499-40-4 | 2 | <LOQ | <LOQ | <LOQ | <LOQ | 4.4 | 2.3 | <LOQ | <LOQ | <LOQ | <LOQ | <LOQ | <LOQ | <LOQ |
| Emtricitabine S-oxide | 152128-77-3 | 20 | <LOQ | <LOQ | <LOQ | <LOQ | <LOQ | <LOQ | <LOQ | <LOQ | <LOQ | <LOQ | <LOQ | <LOQ | <LOQ |
| Emtricitabine-carboxylate | 1238210-10-0 | 10 | <LOQ | <LOQ | <LOQ | <LOQ | 24.8 | 17.0 | 18.7 | 18.2 | 17.4 | 17.4 | 15.2 | 13.8 | 12.4 |
| Flecainide-meta-O-dealkylated | 83526-33-4 | 1 | <LOQ | <LOQ | 4.2 | 3.1 | 11.1 | 8.3 | 8.0 | 6.3 | 5.4 | 4.5 | 3.2 | 2.5 | 2.8 |
| Gabapentin-lactam | 64744-50-9 | 10 | <LOQ | 88.8 | 275.3 | 162.7 | 1437.8 | 1094.9 | 987.4 | 804.2 | 1240.0 | 1117.0 | 922.5 | 698.0 | 683.3 |
| 2-Hydroxy-ibuprofen | 51146-55-5 | 20 | <LOQ | <LOQ | 38.2 | 24.3 | <LOQ | 50.1 | 59.4 | 59.4 | <LOQ | 47.3 | 55.1 | 53.6 | 205.0 |
| Carboxy-ibuprofen | 15935-54-3 | 20 | <LOQ | <LOQ | <LOQ | <LOQ | <LOQ | 20.2 | <LOQ | <LOQ | <LOQ | 53.4 | 41.5 | 44.3 | 127.2 |
| 2-N-methyl-lamotrigine | 1152091-68-3 | 2 | <LOQ | <LOQ | <LOQ | <LOQ | 12.3 | 8.1 | 7.4 | 6.9 | 8.1 | 7.0 | 6.2 | 5.2 | 5.6 |
| Levetiracetam acid | 103833-72-3 | 50 | <LOQ | <LOQ | <LOQ | <LOQ | <LOQ | <LOQ | <LOQ | <LOQ | <LOQ | <LOQ | <LOQ | <LOQ | <LOQ |
| Nor-Lidocaine | 7729-94-4 | 10 | <LOQ | <LOQ | <LOQ | <LOQ | <LOQ | <LOQ | <LOQ | <LOQ | <LOQ | <LOQ | <LOQ | <LOQ | <LOQ |
| Oxypurinol | 2465-59-0 | 50 | <LOQ | 360.0 | 1261.9 | 443.8 | 6492.1 | 4972.3 | 4259.1 | 3528.2 | 3582.8 | 3427.3 | 2286.9 | 2648.2 | 3150.3 |
| α-Hydoxy-metoprolol | 56392-16-6 | 5 | <LOQ | <LOQ | 5.6 | <LOQ | 9.1 | 8.6 | 10.2 | 9.0 | 6.2 | 5.7 | 5.3 | 5.2 | 7.0 |
| O-Desmethyl-metoprolol | 62572-94-5 | 5 | <LOQ | <LOQ | <LOQ | <LOQ | <LOQ | <LOQ | <LOQ | <LOQ | <LOQ | <LOQ | <LOQ | <LOQ | <LOQ |
| O-Desmethyl-naproxen | 52079-10-4 | 5 | <LOQ | <LOQ | <LOQ | <LOQ | <LOQ | <LOQ | <LOQ | <LOQ | <LOQ | <LOQ | <LOQ | <LOQ | <LOQ |
| Ramiprilat | 87269-97-4 | 20 | <LOQ | <LOQ | 41.5 | 20.5 | 44.8 | 51.6 | 71.9 | 16.6 | 26.2 | 29.7 | <LOQ | <LOQ | 34.2 |
| Desmethyl-ranitidine | 66357-25-3 | 5 | <LOQ | <LOQ | <LOQ | <LOQ | <LOQ | <LOQ | <LOQ | <LOQ | <LOQ | <LOQ | <LOQ | <LOQ | <LOQ |
| Ranitidine-N-oxide | 73857-20-2 | 10 | <LOQ | <LOQ | <LOQ | <LOQ | <LOQ | <LOQ | <LOQ | <LOQ | <LOQ | <LOQ | <LOQ | <LOQ | <LOQ |
| Ranitidine-S-oxide | 73851-70-4 | 10 | <LOQ | <LOQ | <LOQ | <LOQ | 94.2 | 63.6 | 55.8 | 43.7 | 31.5 | 26.1 | 21.4 | 17.2 | 15.3 |
| Ritalinic acid | 19395-41-6 | 20 | <LOQ | <LOQ | 308.4 | 204.1 | 630.7 | 558.3 | 557.9 | 485.9 | 463.6 | 415.5 | 388.1 | 342.5 | 368.7 |
| Hydroxy-torasemide | 99300-68-2 | 10 | <LOQ | <LOQ | <LOQ | <LOQ | <LOQ | <LOQ | <LOQ | <LOQ | <LOQ | <LOQ | <LOQ | <LOQ | <LOQ |
| N-Desmethyl-tramadol | 75377-45-6 | 10 | <LOQ | <LOQ | <LOQ | <LOQ | 51.5 | 39.3 | 42.7 | 36.4 | 32.5 | 27.7 | 25.1 | 24.1 | 28.2 |
| O-Desmethyl-tramadol | 80456-81-1 | 5 | <LOQ | 5.5 | 20.5 | 9.3 | 153.6 | 115.4 | 106.3 | 131.6 | 93.6 | 79.0 | 115.7 | 203.4 | 83.6 |
| N,N-Didesmethyl-tramadol | 541505-91-3 | 50 | <LOQ | 50.0 | <LOQ | 50.0 | 50.0 | 87.5 | 89.8 | 87.4 | 84.1 | 82.6 | 79.8 | 72.3 | 50.0 |
| N,O-Didesmethyl-tramadol | 333368-16-2 | 10 | <LOQ | 10.2 | 30.8 | 12.2 | 218.0 | 166.8 | 158.8 | 89.3 | 128.9 | 112.5 | 76.1 | 121.9 | 118.6 |
| Tramadol-N-oxide | 147441-56-3 | 2 | <LOQ | <LOQ | <LOQ | <LOQ | 4.1 | <LOQ | <LOQ | <LOQ | 2.6 | 2.0 | <LOQ | <LOQ | <LOQ |
| 3-Desmethyl-trimethoprim | 27653-69-6 | 5 | <LOQ | <LOQ | <LOQ | <LOQ | <LOQ | <LOQ | <LOQ | <LOQ | <LOQ | <LOQ | <LOQ | <LOQ | <LOQ |
| Valsartan acid | | 10 | <LOQ | 87.0 | 331.6 | 195.7 | 2728.8 | 2090.6 | 1972.1 | 1449.0 | 2629.2 | 2417.9 | 1973.2 | 1260.0 | 1418.7 |
| N-Desmethyl-venlafaxine | 149289-30-5 | 5 | <LOQ | <LOQ | <LOQ | <LOQ | 19.6 | 13.7 | 12.0 | 10.7 | 14.3 | 11.9 | 6.5 | 7.3 | 7.4 |
| N,N-Didesmethyl-venlafaxine | 130198-05-9 | 5 | <LOQ | 5.0 | <LOQ | 5.0 | 5.0 | 22.5 | 20.6 | 19.4 | 18.1 | 17.2 | 15.4 | 10.6 | 5.0 |
| N,O-Desmethyl-venlafaxine | 135308-74-6 | 100 | <LOQ | <LOQ | <LOQ | <LOQ | <LOQ | <LOQ | <LOQ | <LOQ | <LOQ | <LOQ | <LOQ | <LOQ | <LOQ |
| O-Desmethyl-venlafaxine | 93413-62-8 | 5 | <LOQ | 14.8 | 28.6 | 12.3 | 220.7 | 201.9 | 183.4 | 177.9 | 206.5 | 172.9 | 118.0 | 125.7 | 124.5 |
| Venlafaxine-N-oxide | 1094598-37-4 | 1 | <LOQ | <LOQ | <LOQ | <LOQ | <LOQ | <LOQ | <LOQ | <LOQ | <LOQ | <LOQ | <LOQ | <LOQ | <LOQ |
| **Biocides and pesticides** | | | | | | | | | | | | | | | |
| Carbanilid | 102-07-8 | 1 | <LOQ | <LOQ | <LOQ | 1.4 | 2.0 | 1.6 | 2.4 | 2.2 | 2.7 | 3.4 | 3.1 | 2.9 | 2.7 |
| Carbendazim | 10605-21-7 | 5 | <LOQ | <LOQ | <LOQ | <LOQ | 26.3 | 19.3 | 16.2 | 19.5 | 30.5 | 26.5 | 17.1 | 18.6 | 16.5 |
| Climbazole | 38083-17-9 | 5 | <LOQ | <LOQ | <LOQ | <LOQ | 8.4 | 5.6 | 6.2 | 5.4 | <LOQ | <LOQ | <LOQ | <LOQ | <LOQ |
| Diuron (DCMU) | 330-54-1 | 2 | <LOQ | <LOQ | 4.1 | 4.1 | 13.8 | 7.4 | 9.8 | 12.7 | 20.9 | 18.0 | 12.6 | 12.9 | 8.7 |
| Epoxiconazole | 133855-98-8 | 1 | <LOQ | <LOQ | 1.6 | <LOQ | 3.7 | 3.7 | 4.0 | 3.8 | 3.5 | 3.6 | 3.0 | 3.0 | 3.2 |
| Imidacloprid | 105827-78-9 | 10 | <LOQ | <LOQ | <LOQ | <LOQ | 13.1 | 11.9 | 11.2 | 10.9 | 13.8 | 11.0 | 10.2 | <LOQ | <LOQ |
| Irgarol | 28159-98-0 | 1 | <LOQ | <LOQ | <LOQ | <LOQ | <LOQ | <LOQ | <LOQ | <LOQ | <LOQ | <LOQ | <LOQ | <LOQ | <LOQ |
| Isoproturon | 34123-59-6 | 2 | <LOQ | <LOQ | 5.6 | <LOQ | 25.6 | 24.8 | 25.7 | 10.6 | 15.3 | 14.0 | 18.8 | 20.3 | 25.0 |
| Mecoprop | 7085-19-0 | 5 | <LOQ | <LOQ | 9.1 | <LOQ | 8.2 | 15.4 | 14.7 | 15.8 | 10.5 | 15.5 | 15.7 | 12.7 | 7.9 |
| Metamitron | 41394-05-2 | 1 | <LOQ | <LOQ | <LOQ | <LOQ | 2.6 | <LOQ | <LOQ | <LOQ | <LOQ | <LOQ | <LOQ | <LOQ | <LOQ |
| Metazachlor | 67129-08-2 | 1 | <LOQ | 6.7 | 2.1 | 5.6 | 13.2 | 9.0 | 11.4 | 10.8 | 6.0 | 5.3 | 4.8 | 4.5 | 19.1 |
| Metolachlor | 51218-45-2 | 1 | <LOQ | <LOQ | <LOQ | <LOQ | 1.1 | <LOQ | 1.1 | 1.3 | 2.9 | 3.3 | <LOQ | <LOQ | 1.7 |
| Propiconazole | 60207-90-1 | 2 | <LOQ | <LOQ | <LOQ | <LOQ | 3.0 | 3.2 | 6.9 | 6.6 | 4.2 | 3.8 | 5.4 | 4.8 | 2.9 |
| Tebuconazole | 107534-96-3 | 2 | <LOQ | <LOQ | 2.4 | <LOQ | 6.2 | 6.3 | 8.1 | 7.4 | 6.8 | 6.7 | 6.2 | 4.1 | 2.2 |
| Terbuthylazine | 5915-41-3 | 1 | <LOQ | <LOQ | <LOQ | <LOQ | 2.7 | 3.3 | 2.8 | 2.6 | 5.8 | 6.5 | 5.2 | 4.2 | 3.6 |
| Terbutryn | 886-50-0 | 1 | <LOQ | 2.4 | 3.2 | 3.1 | 27.4 | 19.1 | 17.8 | 15.6 | 16.9 | 16.0 | 15.1 | 15.5 | 15.9 |
| Triclocarban | 101-20-2 | 2 | <LOQ | <LOQ | <LOQ | <LOQ | 2.1 | <LOQ | <LOQ | <LOQ | 5.0 | 5.6 | 5.3 | 4.9 | 2.1 |
| Triclosan | 3380-34-5 | 2 | <LOQ | <LOQ | <LOQ | <LOQ | 2.3 | <LOQ | 3.3 | 3.4 | 5.0 | 4.9 | 4.5 | 4.1 | 2.3 |
| **TPs of biocides and pesticides** | | | | | | | | | | | | | | | |
| Climbazol-TP | 55362-18-0 | 20 | <LOQ | <LOQ | <LOQ | <LOQ | 37.3 | 28.8 | 27.2 | <LOQ | 29.4 | 25.8 | <LOQ | <LOQ | 20.2 |
| Desmethyl-diuron (DCPMU) | 3567-62-2 | 1 | <LOQ | <LOQ | <LOQ | <LOQ | <LOQ | <LOQ | 1.0 | 1.0 | 1.3 | 1.5 | 1.5 | 1.5 | 1.7 |
| 3,4-dichlorophenyl-urea (DCPU) | 2327-02-8 | 5 | <LOQ | <LOQ | <LOQ | <LOQ | <LOQ | <LOQ | <LOQ | <LOQ | <LOQ | <LOQ | <LOQ | <LOQ | <LOQ |
| Desamino-metamitron | 36993-94-9 | 5 | <LOQ | <LOQ | <LOQ | <LOQ | <LOQ | <LOQ | <LOQ | <LOQ | <LOQ | <LOQ | <LOQ | <LOQ | <LOQ |
| Metolachlor-ethanesulfonic acid | 171118-09-5 | 2 | 14.5 | 9.2 | 6.8 | 32.3 | 9.5 | 10.7 | 6.9 | 26.1 | 9.8 | 9.7 | 23.5 | 23.7 | 14.9 |
| Metolachlor-oxanillic acid | 152019-73-3 | 10 | <LOQ | <LOQ | <LOQ | <LOQ | <LOQ | <LOQ | <LOQ | <LOQ | <LOQ | <LOQ | <LOQ | <LOQ | <LOQ |
| 2-Hydroxy-terbuthylazine | 66753-07-9 | 5 | <LOQ | <LOQ | <LOQ | <LOQ | 12.7 | 11.0 | 10.8 | 10.3 | 16.4 | 17.3 | 15.6 | 15.8 | 12.4 |
| Terbuthylazine-desethyl | 30125-63-4 | 1 | <LOQ | <LOQ | <LOQ | 3.9 | 6.2 | <LOQ | <LOQ | <LOQ | 7.9 | 7.8 | 2.3 | <LOQ | 6.8 |
| Terbutryn-sulfoxide | 82985-33-9 | 2 | <LOQ | <LOQ | <LOQ | 2.7 | 50.6 | 13.2 | 11.7 | 12.7 | 24.8 | 21.2 | 22.3 | 20.9 | 24.2 |
| **Specific industrial compounds** | | | | | | | | | | | | | | | |
| 4-Chlorobenzoic-acid | 74-11-3 | 5 | <LOQ | <LOQ | <LOQ | <LOQ | <LOQ | <LOQ | <LOQ | <LOQ | <LOQ | <LOQ | <LOQ | <LOQ | <LOQ |
| Ethyltriphenylphosphonium | 1530-32-1 | 1 | <LOQ | <LOQ | <LOQ | <LOQ | <LOQ | <LOQ | <LOQ | <LOQ | 1.0 | 1.0 | 1.0 | 1.0 | <LOQ |
| (Methoxymethyl)triphenylphosphonium | 4009-98-7 | 1 | <LOQ | <LOQ | <LOQ | <LOQ | <LOQ | <LOQ | 1.4 | 1.4 | 1.0 | 1.0 | 1.0 | 1.0 | <LOQ |
| Methyltriphenylphosphonium | 1779-49-3 | 1 | <LOQ | <LOQ | <LOQ | <LOQ | <LOQ | <LOQ | <LOQ | <LOQ | 1.0 | 1.0 | 1.0 | 1.0 | <LOQ |
| Tetrabutylammonium | 2052-49-5 | 10 | <LOQ | <LOQ | <LOQ | <LOQ | <LOQ | <LOQ | <LOQ | <LOQ | 14.1 | 13.7 | 13.4 | 13.1 | <LOQ |
| Tetrabutylphosphonium | 3115-68-2 | 1 | <LOQ | <LOQ | <LOQ | <LOQ | <LOQ | <LOQ | <LOQ | <LOQ | <LOQ | <LOQ | <LOQ | <LOQ | <LOQ |
| Tetraglyme | 143-24-8 | 5 | <LOQ | <LOQ | <LOQ | <LOQ | 18.2 | 15.3 | 14.5 | 16.8 | 23.0 | 24.6 | 24.7 | 21.1 | 16.7 |
| Tetrapropylammonium | 5810-42-4 | 1 | <LOQ | <LOQ | <LOQ | <LOQ | <LOQ | <LOQ | <LOQ | <LOQ | <LOQ | <LOQ | <LOQ | <LOQ | <LOQ |
| o-Tolylbiguanide | 93-69-6 | 10 | <LOQ | <LOQ | <LOQ | <LOQ | 142.1 | 738.4 | 625.5 | 604.8 | 365.5 | 315.4 | 309.7 | 305.1 | 289.0 |
| Triphenyl phosphate | 115-86-6 | 50 | <LOQ | <LOQ | <LOQ | <LOQ | <LOQ | <LOQ | <LOQ | <LOQ | <LOQ | <LOQ | <LOQ | <LOQ | <LOQ |
| **X-ray contrast media** | | | | | | | | | | | | | | | |
| Diatrizoate | 117-96-4 | 20 | <LOQ | 214.0 | 324.8 | 76.1 | 359.7 | 450.7 | 600.8 | 34.9 | 461.8 | 392.8 | 27.0 | 33.5 | 390.6 |
| Iomeprol | 78649-41-9 | 50 | <LOQ | <LOQ | 296.9 | <LOQ | 463.1 | 371.7 | 2093.6 | 50.5 | 207.6 | 194.0 | 159.3 | 165.6 | 294.1 |
| Iopamidol | 60166-93-0 | 50 | <LOQ | <LOQ | <LOQ | <LOQ | <LOQ | 69.6 | 361.0 | <LOQ | <LOQ | <LOQ | <LOQ | <LOQ | <LOQ |
| Iopromide | 73334-07-3 | 50 | <LOQ | <LOQ | <LOQ | <LOQ | 389.9 | 144.9 | 351.3 | 126.3 | 167.2 | 143.1 | 127.7 | 118.0 | 133.0 |
| **Artificial sweeteners** | | | | | | | | | | | | | | | |
| Acesulfame | 55589-62-3 | 50 | <LOQ | 938.0 | 580.2 | 769.6 | 823.6 | 773.7 | 755.6 | 479.2 | 440.7 | 767.9 | 757.6 | 728.6 | 998.1 |
| Saccharin | 81-07-2 | 10 | 15.2 | 37.0 | 56.3 | 97.9 | 53.8 | 72.2 | 180.4 | 66.6 | 50.9 | 71.4 | 195.8 | 155.3 | 149.3 |

| | | | | | | | | | | | | | | | |
|---|---|---|---|---|---|---|---|---|---|---|---|---|---|---|---|
| Sucralose | 56038-13-2 | 200 | <LOQ | <LOQ | 454.0 | 225.1 | 1362.8 | 1260.0 | 1254.7 | 862.8 | 1360.3 | 1306.3 | 991.5 | 819.0 | 774.1 |
| **Corrosion inhibitors and their TPs** | | | | | | | | | | | | | | | |
| Benzotriazole | 95-14-7 | 20 | <LOQ | 168.0 | 425.4 | 202.8 | 1502.1 | 1241.9 | 1296.1 | 991.6 | 933.3 | 888.8 | 816.9 | 763.3 | 886.0 |
| 1-Hydroxy-benzotriazole | 2592-95-2 | 20 | <LOQ | <LOQ | <LOQ | <LOQ | 28.7 | 26.3 | 20.8 | 17.4 | 15.2 | 14.9 | 13.3 | 12.6 | 11.9 |
| 4-Hydroxy-benzotriazole | 26725-51-9 | 50 | <LOQ | <LOQ | <LOQ | <LOQ | <LOQ | <LOQ | <LOQ | <LOQ | <LOQ | <LOQ | <LOQ | <LOQ | <LOQ |
| Tolyltriazole | 29385-43-1 | 20 | <LOQ | 75.0 | 66.0 | 110.0 | 1300.0 | 1400.0 | 1200.0 | 1200.0 | 1200.0 | 1200.0 | 1200.0 | 1200.0 | 1400.0 |
| **Repellents and their TPs** | | | | | | | | | | | | | | | |
| Diethyltoluamide (DEET) | 134-62-3 | 5 | <LOQ | 11.5 | 19.4 | 16.3 | 117.8 | 57.3 | 72.0 | 85.5 | 128.9 | 100.8 | 71.5 | 67.2 | 64.3 |
| DEET carboxylic acid | 72236-23-8 | 2 | <LOQ | <LOQ | 3.9 | 3.1 | 144.8 | 77.4 | 79.2 | 104.9 | 154.7 | 129.8 | 94.7 | 55.7 | 63.0 |
| Hydroxy-DEET | 72236-22-7 | 1 | <LOQ | <LOQ | <LOQ | <LOQ | <LOQ | <LOQ | <LOQ | <LOQ | <LOQ | <LOQ | <LOQ | <LOQ | <LOQ |
| N-ethyl-m-toluamid | 26819-07-8 | 10 | <LOQ | <LOQ | <LOQ | <LOQ | <LOQ | <LOQ | <LOQ | <LOQ | <LOQ | <LOQ | <LOQ | <LOQ | <LOQ |
| **Aversive agent** | | | | | | | | | | | | | | | |
| Denatonium | 3734-33-6 | 10 | <LOQ | <LOQ | <LOQ | <LOQ | 40.5 | 24.4 | 22.2 | 19.5 | 30.2 | 25.3 | 38.8 | 17.9 | 14.7 |
| **Stimulants** | | | | | | | | | | | | | | | |
| Caffeine | 58-08-2 | 100 | <LOQ | 112.5 | 317.4 | <LOQ | <LOQ | <LOQ | 383.1 | 283.4 | <LOQ | 176.4 | 226.5 | 206.3 | 322.2 |

**Table S4** Calculated toxic units (TUs) for 57 of the 161 analysed compounds from table S2 with available toxicity data in acute tests with *Daphnia* spp. ($EC_{50}$ values). The mixture effect ($E_{CA}$) for the sampling sites is calculated as the sum TUs.

| | | | | Toxic units (TUs) at sampling sites | | | | | | | | | | | | |
|---|---|---|---|---|---|---|---|---|---|---|---|---|---|---|---|---|
| Chemical | CAS_NO | $EC_{50}$ *Daphnia* (mg/l) | Source | I | Ia | II | III | IIIa | IV | IVa | IVb | ÎVc | V | Va | Vb | VI |
| **Pharmaceuticals** | | | | | | | | | | | | | | | | |
| Acetaminophen | 103-90-2 | 11.85 | ECOTOX | 0 | 0 | 0 | 0 | 0 | 0 | 0 | 0 | 0 | 0 | 0 | 0 | 0 |
| Atenolol | 29122-68-7 | 313 | Cleuvers (2005) | 0 | 0 | 0 | 0 | 0 | 0 | 0 | 0 | 0 | 0 | 0 | 0 | 0 |
| Bezafibrate | 41859-67-0 | 30.3 | ECOTOX | 0 | 0 | 5.96E-07 | 1.68E-07 | 4.21E-07 | 5.61E-07 | 6.63E-07 | 5.38E-07 | 0 | 0 | 1.90E-07 | 1.91E-07 | 2.67E-07 |
| Candesartan | 139481-59-7 | 120 | ECHA | 0 | 1.18E-07 | 7.88E-07 | 2.67E-07 | 3.29E-06 | 2.93E-06 | 2.79E-06 | 2.59E-06 | 3.05E-06 | 2.88E-06 | 1.34E-06 | 1.53E-06 | 1.76E-06 |
| Carbamazepine | 298-46-4 | 111 | ECOTOX | 0 | 0 | 4.54E-07 | 1.76E-07 | 2.01E-06 | 1.71E-06 | 1.68E-06 | 1.66E-06 | 1.52E-06 | 1.41E-06 | 5.94E-07 | 6.76E-07 | 1.16E-06 |
| Citalopram | 59729-33-8 | 3.9 | ECOTOX | 0 | 0 | 1.53E-06 | 0 | 9.44E-06 | 3.02E-06 | 1.97E-06 | 1.90E-06 | 1.84E-06 | 1.37E-06 | 1.33E-06 | 1.31E-06 | 1.83E-06 |
| Diclofenac | 15307-86-5 | 56.6 | ECOTOX | 0 | 0 | 2.45E-06 | 8.41E-07 | 9.48E-06 | 7.53E-06 | 6.30E-06 | 6.28E-06 | 3.97E-06 | 4.17E-06 | 4.12E-06 | 4.23E-06 | 3.30E-06 |
| Diphenhydramine | 58-73-1 | 3.94 | ECOTOX | 0 | 0 | 3.50E-07 | 2.79E-07 | 3.27E-06 | 9.00E-07 | 5.88E-07 | 5.46E-07 | 3.81E-07 | 3.55E-07 | 4.44E-07 | 4.44E-07 | 2.79E-07 |
| Erythromycin | 114-07-8 | 24 | ECOTOX | 0 | 0 | 0 | 0 | 3.74E-07 | 2.53E-07 | 2.33E-07 | 2.96E-07 | 4.00E-07 | 2.76E-07 | 2.42E-07 | 2.33E-07 | 0 |
| Ibuprofen | 15687-27-1 | 0.032 | ECOTOX | 0 | 0 | 0 | 0 | 0 | 0 | 0 | 0 | 0 | 0 | 0 | 0 | 1.45E-03 |
| Lidocaine | 137-58-6 | 309 | Lomba et al. (2020) | 0 | 1.43E-08 | 5.69E-08 | 2.26E-08 | 1.20E-07 | 1.14E-07 | 1.08E-07 | 8.38E-08 | 8.02E-08 | 6.93E-08 | 7.02E-08 | 7.35E-08 | 5.97E-08 |
| Metoprolol | 37350-58-6 | 63.9 | Huggett et al. (2002) | 0 | 2.54E-07 | 6.79E-07 | 2.33E-07 | 2.22E-06 | 1.69E-06 | 1.81E-06 | 1.21E-06 | 1.12E-06 | 9.57E-07 | 8.54E-07 | 7.70E-07 | 9.94E-07 |
| Naproxen | 22204-53-1 | 0.032 | ECOTOX | 0 | 0 | 6.90E-04 | 0 | 0 | 7.18E-04 | 1.25E-03 | 7.06E-04 | 0 | 0 | 0 | 0 | 0 |
| Ranitidine | 66357-35-5 | 247 | Godoy et al. (2020) | 0 | 0 | 0 | 0 | 1.31E-07 | 7.24E-08 | 0 | 0 | 0 | 0 | 0 | 0 | 0 |
| Sotalol | 3930-20-9 | 325 | Godoy et al. (2020) | 0 | 0 | 4.43E-08 | 0 | 1.37E-07 | 1.18E-07 | 1.12E-07 | 9.11E-08 | 9.57E-08 | 8.16E-08 | 8.86E-08 | 8.92E-08 | 8.02E-08 |
| Sulfamethoxazole | 723-46-6 | 234 | ECOTOX | 0 | 0 | 7.06E-08 | 1.99E-08 | 3.84E-07 | 2.38E-07 | 2.49E-07 | 2.75E-07 | 5.33E-07 | 4.61E-07 | 2.82E-07 | 2.84E-07 | 2.20E-07 |
| Sulpiride | 15676-16-1 | 21.2 | ECHA | 0 | 0 | 0 | 0 | 3.53E-06 | 2.40E-06 | 2.03E-06 | 2.26E-06 | 2.50E-06 | 2.05E-06 | 1.91E-06 | 1.48E-06 | 1.63E-06 |
| Tramadol | 27203-92-5 | 170 | Le et al. (2011) | 2.65E-08 | 3.55E-08 | 1.59E-07 | 6.75E-08 | 1.12E-06 | 8.37E-07 | 7.91E-07 | 7.08E-07 | 6.53E-07 | 5.69E-07 | 5.16E-07 | 6.06E-07 | 5.58E-07 |
| Trimethoprim | 738-70-5 | 123 | Halling-Sorensen et al. (2000) | 0 | 0 | 6.81E-08 | 1.02E-07 | 1.24E-07 | 7.16E-08 | 9.47E-08 | 8.05E-08 | 7.04E-08 | 5.84E-08 | 5.49E-08 | 5.00E-08 | 4.25E-08 |
| Venlafaxine | 93413-69-5 | 141 | Minguez et al. (2014) | 0 | 2.57E-08 | 8.12E-08 | 2.90E-08 | 7.57E-07 | 4.69E-07 | 3.87E-07 | 3.81E-07 | 4.24E-07 | 3.52E-07 | 2.96E-07 | 2.79E-07 | 2.40E-07 |
| **Transformation products (TPs) of pharmaceuticals** | | | | | | | | | | | | | | | | |
| Chloraminophenamide | 121-30-2 | 837 | ECHA | 0 | 0 | 9.26E-08 | 3.29E-08 | 4.72E-07 | 3.34E-07 | 3.04E-07 | 3.33E-07 | 5.17E-07 | 4.12E-07 | 3.12E-07 | 2.32E-07 | 1.79E-07 |
| **Biocides and pesticides** | | | | | | | | | | | | | | | | |
| Carbendazim | 10605-21-7 | 0.0229 | ECOTOX | 0 | 0 | 0 | 0 | 1.15E-03 | 8.44E-04 | 7.06E-04 | 8.52E-04 | 1.33E-03 | 1.16E-03 | 7.47E-04 | 8.12E-04 | 7.21E-04 |
| Climbazole | 38083-17-9 | 16 | Richter et al. (2013) | 0 | 0 | 0 | 0 | 5.26E-07 | 3.51E-07 | 3.90E-07 | 3.38E-07 | 0 | 0 | 0 | 0 | 0 |
| Diuron (DCMU) | 330-54-1 | 0.4 | ECOTOX | 0 | 0 | 1.02E-05 | 1.02E-05 | 3.46E-05 | 1.84E-05 | 2.45E-05 | 3.18E-05 | 5.22E-05 | 4.49E-05 | 3.15E-05 | 3.23E-05 | 2.17E-05 |
| Epoxiconazole | 133855-98-8 | 5.24 | ECOTOX | 0 | 0 | 3.15E-07 | 0 | 7.08E-07 | 7.12E-07 | 7.61E-07 | 7.25E-07 | 6.77E-07 | 6.83E-07 | 5.73E-07 | 5.73E-07 | 6.08E-07 |
| Imidacloprid | 105827-78-9 | 85 | IUPAC-PPDB | 0 | 0 | 0 | 0 | 1.54E-07 | 1.40E-07 | 1.32E-07 | 1.28E-07 | 1.62E-07 | 1.29E-07 | 1.20E-07 | 0 | 0 |
| Mecoprop | 7085-19-0 | 200 | IUPAC-PPDB | 0 | 0 | 4.57E-08 | 0 | 4.12E-08 | 7.71E-08 | 7.35E-08 | 7.90E-08 | 5.23E-08 | 7.76E-08 | 7.85E-08 | 6.35E-08 | 3.97E-08 |
| Metamitron | 41394-05-2 | 5.7 | IUPAC-PPDB | 0 | 0 | 0 | 0 | 4.52E-07 | 0 | 0 | 0 | 0 | 0 | 0 | 0 | 0 |
| Metazachlor | 67129-08-2 | 33 | IUPAC-PPDB | 0 | 2.04E-07 | 6.24E-08 | 1.70E-07 | 3.98E-07 | 2.74E-07 | 3.46E-07 | 3.27E-07 | 1.82E-07 | 1.61E-07 | 1.45E-07 | 1.36E-07 | 5.77E-07 |
| Metolachlor | 51218-45-2 | 13 | ECOTOX | 0 | 0 | 0 | 0 | 8.65E-08 | 0 | 8.28E-08 | 1.00E-07 | 2.23E-07 | 2.57E-07 | 0 | 0 | 1.34E-07 |
| Propiconazole | 60207-90-1 | 0.18 | ECOTOX | 0 | 0 | 0 | 0 | 1.68E-05 | 1.80E-05 | 3.86E-05 | 3.67E-05 | 2.35E-05 | 2.09E-05 | 2.97E-05 | 2.67E-05 | 1.60E-05 |
| Tebuconazole | 107534-96-3 | 0.75 | ECOTOX | 0 | 0 | 3.15E-06 | 0 | 8.33E-06 | 8.40E-06 | 1.08E-05 | 9.87E-06 | 9.01E-06 | 8.98E-06 | 8.27E-06 | 5.47E-06 | 2.91E-06 |
| Terbuthylazine | 5915-41-3 | 21.2 | IUPAC-PPDB | 0 | 0 | 0 | 0 | 1.25E-07 | 1.57E-07 | 1.33E-07 | 1.23E-07 | 2.72E-07 | 3.05E-07 | 2.45E-07 | 1.98E-07 | 1.69E-07 |
| Terbutryn | 886-50-0 | 2.66 | IUPAC-PPDB | 0 | 8.95E-07 | 1.21E-06 | 1.17E-06 | 1.03E-05 | 7.17E-06 | 6.70E-06 | 5.86E-06 | 6.35E-06 | 6.01E-06 | 5.68E-06 | 5.83E-06 | 5.97E-06 |
| Triclocarban | 101-20-2 | 0.0077 | ECOTOX | 0 | 0 | 0 | 0 | 2.73E-04 | 0 | 0 | 0 | 6.49E-04 | 7.32E-04 | 6.88E-04 | 6.36E-04 | 2.78E-04 |

| | | | | | | | | | | | | | | | | |
|---|---|---|---|---|---|---|---|---|---|---|---|---|---|---|---|---|
| Triclosan | 3380-34-5 | 0.115 | ECOTOX | 0 | 0 | 0 | 0 | 2.03E-05 | 0 | 2.86E-05 | 2.96E-05 | 4.35E-05 | 4.26E-05 | 3.91E-05 | 3.57E-05 | 2.03E-05 |
| **TPs of biocides and pesticides** | | | | | | | | | | | | | | | | |
| Desmethyl-diuron (DCPMU) | 3567-62-2 | 49 | IUPAC-PPDB | 0 | 0 | 0 | 0 | 0 | 0 | 1.99E-08 | 1.99E-08 | 2.58E-08 | 3.12E-08 | 3.12E-08 | 3.12E-08 | 3.50E-08 |
| 3,4-dichlorophenyl-urea (DCPU) | 2327-02-8 | 14 | IUPAC-PPDB | 0 | 0 | 0 | 0 | 0 | 0 | 0 | 0 | 0 | 0 | 0 | 0 | 0 |
| Metolachlor-oxanillic acid | 152019-73-3 | 16.6 | IUPAC-PPDB | 0 | 0 | 0 | 0 | 0 | 0 | 0 | 0 | 0 | 0 | 0 | 0 | 0 |
| 2-Hydroxy-terbuthylazine | 66753-07-9 | 2.8 | IUPAC-PPDB | 0 | 0 | 0 | 0 | 4.53E-06 | 3.93E-06 | 3.84E-06 | 3.68E-06 | 5.85E-06 | 6.17E-06 | 5.57E-06 | 5.64E-06 | 4.44E-06 |
| **Specific industrial compounds** | | | | | | | | | | | | | | | | |
| 4-Chlorobenzoic-acid | 74-11-3 | 100 | ECOTOX | 0 | 0 | 0 | 0 | 0 | 0 | 0 | 0 | 0 | 0 | 0 | 0 | 0 |
| Ethyltriphenylphosphonium | 1530-32-1 | 16.9 | ECHA | 0 | 0 | 0 | 0 | 0 | 0 | 0 | 0 | 5.92E-08 | 5.92E-08 | 5.92E-08 | 5.92E-08 | 0 |
| (Methoxymethyl)triphenylphosphonium | 4009-98-7 | 1.5 | ECHA | 0 | 0 | 0 | 0 | 0 | 0 | 9.14E-07 | 9.14E-07 | 6.67E-07 | 6.67E-07 | 6.67E-07 | 6.67E-07 | 0 |
| Methyltriphenylphosphonium | 1779-49-3 | 2.4 | ECHA | 0 | 0 | 0 | 0 | 0 | 0 | 0 | 0 | 4.17E-07 | 4.17E-07 | 4.17E-07 | 4.17E-07 | 0 |
| Tetrabutylammonium | 2052-49-5 | 16.5 | ECHA | 0 | 0 | 0 | 0 | 0 | 0 | 0 | 0 | 8.54E-07 | 8.32E-07 | 8.12E-07 | 7.94E-07 | 0 |
| Tetrabutylphosphonium | 3115-68-2 | 4.1 | ECHA | 0 | 0 | 0 | 0 | 0 | 0 | 0 | 0 | 0 | 0 | 0 | 0 | 0 |
| Tetraglyme | 143-24-8 | 7467 | ECHA | 0 | 0 | 0 | 0 | 2.43E-09 | 2.05E-09 | 1.94E-09 | 2.25E-09 | 3.08E-09 | 3.29E-09 | 3.31E-09 | 2.83E-09 | 2.24E-09 |
| o-Tolylbiguanide | 93-69-6 | 15 | ECHA | 0 | 0 | 0 | 0 | 9.47E-06 | 4.92E-05 | 4.17E-05 | 4.03E-05 | 2.44E-05 | 2.10E-05 | 2.06E-05 | 2.03E-05 | 1.93E-05 |
| Triphenyl phosphate | 115-86-6 | 0.09 | ECOTOX | 0 | 0 | 0 | 0 | 0 | 0 | 0 | 0 | 0 | 0 | 0 | 0 | 0 |
| **Artificial sweeteners** | | | | | | | | | | | | | | | | |
| Acesulfame | 55589-62-3 | 1000 | ECHA | 0 | 9.38E-07 | 5.80E-07 | 7.70E-07 | 8.24E-07 | 7.74E-07 | 7.56E-07 | 4.79E-07 | 4.41E-07 | 7.68E-07 | 7.58E-07 | 7.29E-07 | 9.98E-07 |
| Saccharin | 81-07-2 | 1000 | ECHA | 1.52E-08 | 3.70E-08 | 5.63E-08 | 9.79E-08 | 5.38E-08 | 7.22E-08 | 1.80E-07 | 6.66E-08 | 5.09E-08 | 7.14E-08 | 1.96E-07 | 1.55E-07 | 1.49E-07 |
| Sucralose | 56038-13-2 | 1800 | ECHA | 0 | 0 | 2.52E-07 | 1.25E-07 | 7.57E-07 | 7.00E-07 | 6.97E-07 | 4.79E-07 | 7.56E-07 | 7.26E-07 | 5.51E-07 | 4.55E-07 | 4.30E-07 |
| **Corrosion inhibitors** | | | | | | | | | | | | | | | | |
| Benzotriazole | 95-14-7 | 102 | ECOTOX | 0 | 1.65E-06 | 4.17E-06 | 1.99E-06 | 1.47E-05 | 1.22E-05 | 1.27E-05 | 9.72E-06 | 9.15E-06 | 8.71E-06 | 8.01E-06 | 7.48E-06 | 8.69E-06 |
| Tolyltriazole | 29385-43-1 | 102 | ECOTOX | 0 | 7.35E-07 | 6.47E-07 | 1.08E-06 | 1.27E-05 | 1.37E-05 | 1.18E-05 | 1.18E-05 | 1.18E-05 | 1.18E-05 | 1.18E-05 | 1.18E-05 | 1.37E-05 |
| **Repellent** | | | | | | | | | | | | | | | | |
| Diethyltoluamide (DEET) | 134-62-3 | 26 | ECOTOX | 0 | 4.43E-07 | 7.46E-07 | 6.28E-07 | 4.53E-06 | 2.20E-06 | 2.77E-06 | 3.29E-06 | 4.96E-06 | 3.88E-06 | 2.75E-06 | 2.58E-06 | 2.47E-06 |
| **Aversive agent** | | | | | | | | | | | | | | | | |
| Denatonium | 3734-33-6 | 500 | ECHA | 0 | 0.00E+00 | 0.00E+00 | 0.00E+00 | 8.09E-08 | 4.89E-08 | 4.43E-08 | 3.90E-08 | 6.05E-08 | 5.06E-08 | 7.75E-08 | 3.58E-08 | 2.93E-08 |
| **Stimulants** | | | | | | | | | | | | | | | | |
| Caffeine | 58-08-2 | 178 | ECOTOX | 0 | 6.32E-07 | 1.78E-06 | 0 | 0 | 0 | 2.15E-06 | 1.59E-06 | 0 | 9.91E-07 | 1.27E-06 | 1.16E-06 | 1.81E-06 |
| | | | **$E_{CA}$ = sum of TUs** | **0.000000** | **0.000006** | **0.000721** | **0.000018** | **0.001598** | **0.001721** | **0.002163** | **0.001765** | **0.002192** | **0.002086** | **0.001617** | **0.001620** | **0.002579** |

## References Appendix A


Cleuvers M (2005): Initial risk assessment for three beta-blockers found in the aquatic environment. Chemosphere 59, 199-205.

Godoy AA, Domingues I, de Carvalho LB, Oliveira AC, de Jesus Azevedo CC, Taparo JM, Assano PK, Mori V, de Almeida Vergara Hidalgo V, Nogueira AJA, Kummrow F (2020): Assessment of the ecotoxicity of the pharmaceuticals bisoprolol, sotalol, and ranitidine using standard and behavioral endpoints. Environ Sci Pollut Res 27, 5469–5481.

Halling-Sorensen B, Holten Lutzhoft HC, Andersen HR, Ingerslev F (2000): Environmental risk assessment of antibiotics: Comparison of mecillinam, trimethoprim and ciprofloxacin. J. Antimicrob. Chemother. 46, 53-58.

Huggett DB, Brooks BW, Peterson B, Foran CM, Schlenk D (2002): Toxicity of select beta adrenergic receptor-blocking pharmaceuticals (ß-blockers) on aquatic organisms. Arch. Environ. Contam. Toxicol. 43, 229-235.

Le TH, Lim ES, Lee SK, Park JS, Kim YH, Min J (2011): Toxicity evaluation of verapamil and tramadol based on toxicity assay and expression patterns of Dhb, Vtg, Arnt, CYP4, and CYP314 in *Daphnia magna*. Environ Toxicol. 26, 515-523.

Lomba L, Lapeña D, Ros N, Aso E, Cannavò M, Errazquin D, Giner B (2020): Ecotoxicological study of six drugs in Aliivibrio fischeri, Daphnia magna and *Raphidocelis subcapitata*. Environ Sci Pollut Res 27, 9891–9900.

Minguez L, Farcy E, Ballandonne C, Lepailleur A, Serpentini A, Lebel JM, Bureau R, Halm-Lemeille MP (2014): Acute toxicity of 8 antidepressants: What are their modes of action? Chemosphere 108, 314-319.

Richter E, Wick A, Ternes TA, Coors A (2013): Ecotoxicity of climbazole, a fungicide contained in antidandruff shampoo. Environ. Toxicol. Chem. 32, 2816-2825.

# Appendix B – Molecular identification of parasites

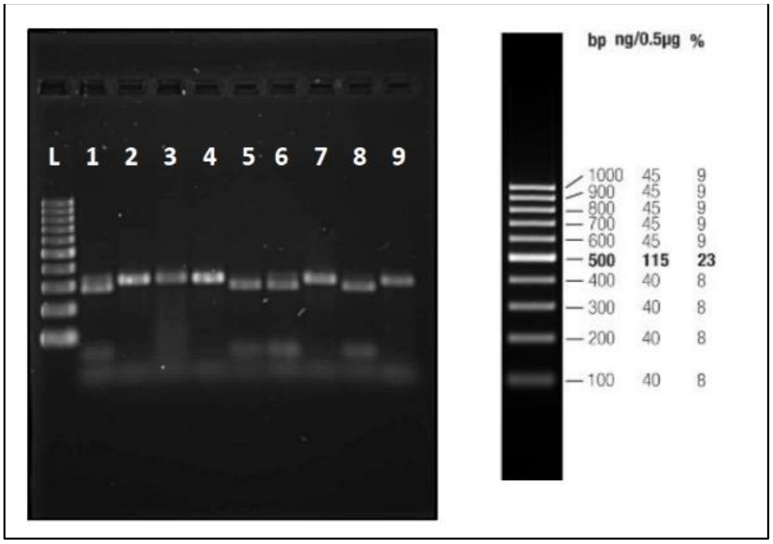


**Figure S3** 1% agarose gel with ladder (L) and samples (1-9). Shorter product bands (1, 5, 6, 8) represent *Polymorphus minutus* samples and longer product bands (2, 3, 4, 7, 9) represent *Pomphorhynchus laevis* samples.

DNA was extracted using the Qiagen DNeasy ® Blood & Tissue Kit according to the instructions. Only 60 µL AE buffer instead of 200 µL were used for elution. In addition to the extraction of individual samples, two 96-well plate extractions were performed according to the "Glass Fiber Plate DNA Extraction" protocol from the Canadian Center for DNA Barcoding (CCDB). All samples for molecular parasite identification were randomly selected, including samples from all sites where infected *G. roeselii* had been found except H3. All acanthocephalans collected from H19 were analysed.

After extraction, a polymerase chain reaction (PCR) was performed for DNA amplification. Each PCR reaction mix (total volume 25 µL) contained 1 µL template DNA, 12.5 µL Taq PCR Master Mix (QIAGEN), 1 µL of each primer, and 9.5 µL purified water. In some cases, a 50 µL PCR mix was used. BD1f (5'GTCGTAACAAGGTTTCCGTA3') and AC/ITS1r(5'TTGCGAGCCAAGTGATTCAC3') were used as primers (Perrot-Minnot 2004; Franceschi et al. 2008). PCR cycling conditions were as follows: initial denaturation for 2 min at 94°C, followed by 40 cycles of 20 sec at 94°C (denaturation), 20 sec at 51°C (annealing), and 50 sec at 72°C (elongation), and finally 5 min at 72°C for the final elongation. The PCR product was then cooled down to 10°C. PCR products were all run on a 1% agarose gel prepared with TAE

buffer and stained with GelRed ® Nucleic Acid Stain (Kisker Biotech GmbH & Co. KG, Steinfurt, Germany). The FastRuler Low Range DNA Ladder (Thermo Fisher Scientific Inc., Waltham, USA) was used as ladder. After gel electrophoresis, gels were irradiated with UV light in the Gel Documentation System Imager Chemi (VWR, Radnor, USA). Photographs of the gels were taken using the GenoCapture program (version number: 7.12.09.0, developer: VWR, Radnor, USA).

Originally, we intended to differentiate between *P. laevis* and *P. tereticollis*, two species that cannot be identified morphologically without ambiguities, and expected a product band of 320 bp and 350 bp for *P. laevis* and *P. tereticollis*, respectively, based on results by Perrot-Minnot (2004) and Franceschi et al. (2008). This procedure was chosen to avoid high sequencing costs. However, we detected no differences between product bands of samples morphologically identified as *P. laevis*, suggesting that no *P. tereticollis* were collected. Individuals that we had previously phenotypically identified as *P. minutus* showed shorter bands (we had no prior information on this). In order to validate these differences in the bands with barcoding data and thus establish a clear link to the species, we sequenced these samples.

Therefore, individual samples were purified using the NucleoSpin ® Gel and PCR Clean-up (Macherey-Nagel, Düren, Germany) according to their instructions. Purification of samples on the two 96-well plates was performed by Microsynth AG (Balgach, Switzerland). The samples on the two 96-well plates and all purified individual samples were sequenced by Microsynth AG (Balgach, Switzerland). Base sequences were edited using the BioEdit Sequence Alignment Editor program (Version 7.2.5). Using the Basic Local Alignment Search Tool BLAST (National Center for Biotechnology Information), the sequences were compared with available sequences in the database. Obtained sequence information ultimately verified the identity of *P. laevis* and *P. minutus*. Only sequences that were > 200 bp long were evaluated.

## References Appendix B

Franceschi N, Bauer A, Bollache L, et al (2008) The effects of parasite age and intensity on variability in acanthocephalan-induced behavioural manipulation. Int J Parasitol 38:1161-1170. 10.1016/j.ijpara.2008.01.003

Perrot-Minnot M-J (2004) Larval morphology, genetic divergence, and contrasting levels of host manipulation between forms of *Pomphorhynchus laevis* (Acanthocephala). Int J Parasitol 34:45–54. https://doi.org/10.1016/j.ijpara.2003.10.005

# Appendix C – Acute toxicity test

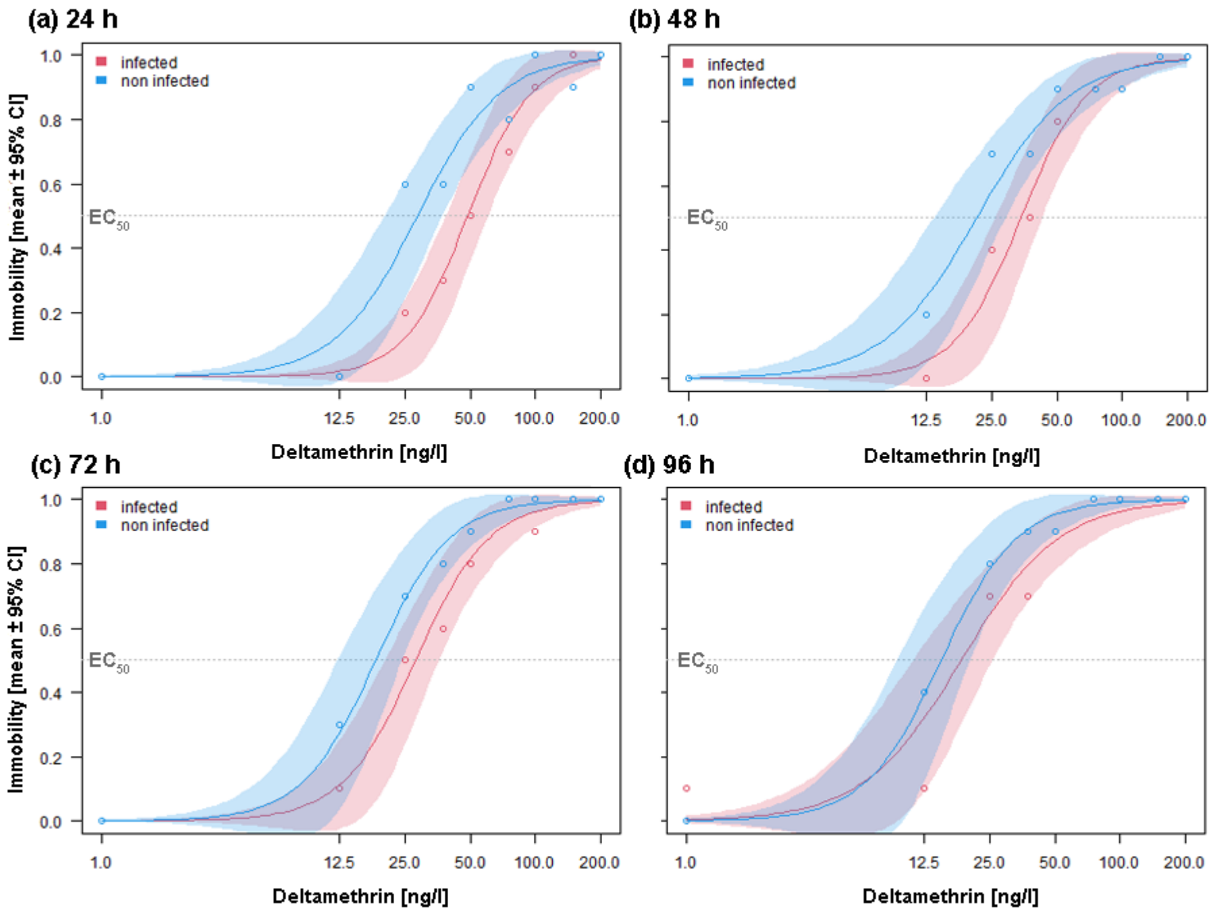


**Figure S4** Sigmoidal curves of two parameter log–logistic models (LL.2) of *G. roeselii* following a (a) 24 h, (b) 48 h, (c) 72 h and (d) 96 h exposure to deltamethrin. Results are shown for uninfected amphipods (blue) and uninfected amphipods (red). Shown are mean immobility values with 95% CI.
